# Semisynthesis of Stable Isotope-Labeled Ergot Alkaloids for HPLC-MS/MS Analysis

**DOI:** 10.1021/acs.jafc.5c03345

**Published:** 2025-07-11

**Authors:** Sven-Oliver Herter, Hajo Haase, Matthias Koch

**Affiliations:** † Division 1.7 Organic Trace and Food Analysis, Bundesanstalt für Materialforschung und -prüfung (BAM), Richard-Willstätter-Str. 11, 12489 Berlin, Germany; ‡ Department of Food Chemistry and Toxicology, Technische Universität Berlin, Gustav-Meyer-Allee 25, 13355 Berlin, Germany

**Keywords:** ergot alkaloids, stable isotope labeling, semisynthesis, HPLC, mass spectrometry

## Abstract

Ergot alkaloids (EAs) are prevalent food contaminants affecting cereals, such as rye, wheat, and barley worldwide. To ensure EU safety standards, the six most common EAs: ergometrine, ergotamine, ergosine, ergocornine, ergocristine, and ergocryptine, and their epimers, are quantified using HPLC-MS/MS, as described in the European Standard Method EN 17425:2021. However, this can be challenging and time-consuming in food matrices without appropriate internal standards and highlights the need for more robust and precise analytical tools to support their monitoring. The development of isotope-labeled EAs directly addresses this gap, offering improved accuracy and leading to more consistency across laboratories and consequently to more consumer safety. Therefore, we developed a semisynthetic approach, building upon our previous work where native ergotamine was *N* ^6^-demethylated to norergotamine and subsequently remethylated using iodomethane (^13^CD_3_-I). Herein, we are now able to present the successful synthesis of all of the isotopically labeled priority EAs. These isotope-labeled standards were tested against their native counterparts using HPLC coupled with HR-MS/MS. The chromatographic and mass spectrometric properties of the unlabeled and isotopically labeled EAs match exactly, confirming their successful synthesis and structure. These standards can now be utilized to enhance the accuracy and reliability of EA quantification in food and feed.

## Graphical abstract

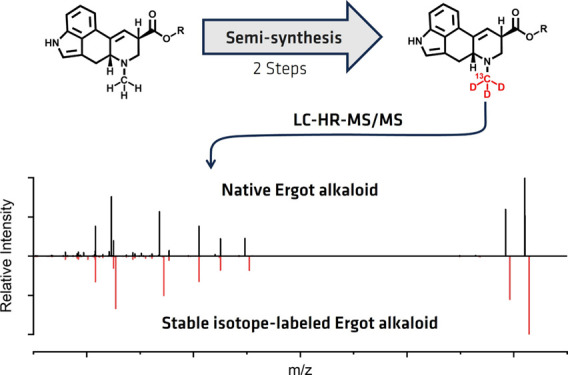

## Introduction

Ergot alkaloids (EAs) are a group of naturally occurring toxic alkaloids produced by fungi of the genus , with being the most prevalent in Europe.^1^ They grow on various cereals, but primarily on rye, wheat, and triticale.^2,3^ When a floret is infected with a spore of ergot fungi, the spore mimics a pollen grain growing into the ovary during fertilization, thus destroying the plant ovary and connecting to the vascular bundle of the plant. The ergot fungi produce asexual spores that are released with a sugary honeydew and dispersed by insects to other florets. In preparation for winter, a hardened mycelium (sclerotia) is formed inside the husk of the floret, and alkaloids and lipids are accumulated in the sclerotium.^4^ The harvest of infected cereal leads to the introduction of sclerotia into the food chain. Mechanical techniques for the removal of sclerotia from grain are effective; however, they do not eliminate contamination completely. Residual sclerotia, in the form of dust or fragments, are ground with grain to flour and thereby introduce toxic EAs into foodstuffs.^4,5^ A defining characteristic of EAs is the tetracyclic ring structure, known as ergoline (Figure 1). Naturally occurring EAs diverge structurally from ergoline due to the methylation of the *N* ^6^-atom and various substituents attached to the *C* ^8^-atom.^6^ Additionally, most EAs possess a double bond between the *C* ^9^- and *C* ^10^-atoms in the ergoline structure.^7^ They can be divided into three different groups based on their structural characteristics: lysergic acid and its simple amides, the ergopeptines, and the ergoclavines.^6^ They all share an *R*-configuration at the *C* ^8^-position of the ergoline structure. Epimerization at the *C* ^8^-atom of simple lysergic acid amides and ergopeptines via keto–enol tautomerism is observed when these EAs are exposed to heat, UV light, or high/low pH.^8,9^ However, ergoclavines, such as lysergine and lysergole, do not undergo epimerization due to the absence of a carboxyl group at the *C* ^8^-atom.

**Figure 1:**
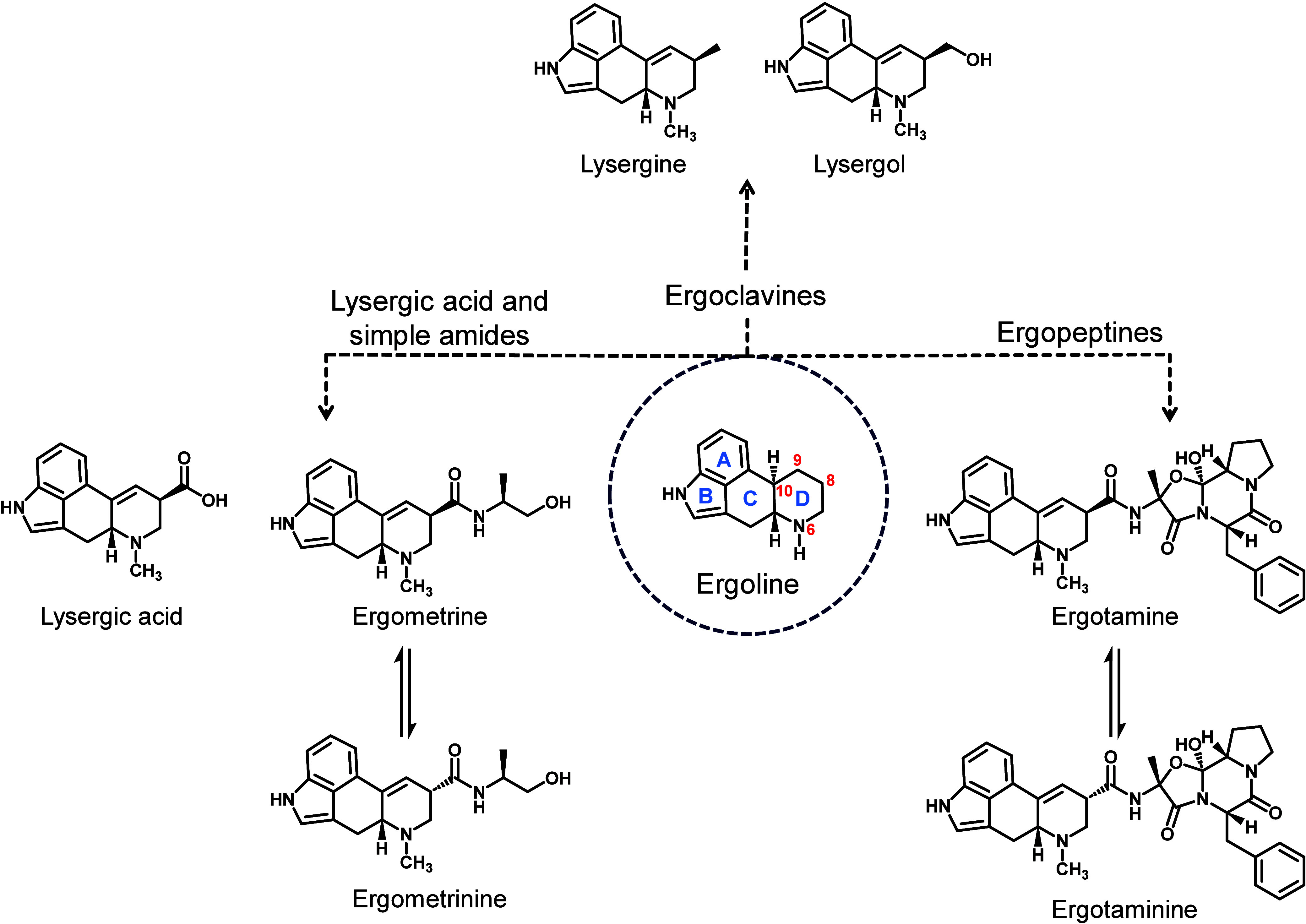
The basic framework of ergoline shows the tetracyclic ring structure and important locants within the molecule. The ergoline structure gives rise to the three groups of ergot alkaloids: ergoclavines, lysergic acid and its simple amides, and ergopeptines. Epimerization can occur at the *C* ^8^-position, resulting in *8R*- and *8S*-configurations of the simple lysergic acid amides and ergopeptines through keto–enol tautomerism.

The simple lysergic acid derivatives include, e.g., the naturally occurring ergometrine and its *8S*-epimer ergometrinine, as well as the semisynthetic drug lysergic acid diethylamide (LSD). Ergopeptines have a more intricate structure, comprising a cyclic tripeptide ring formed by three proteinogenic amino acids.^10,11^ This cyclol-lactam structure is composed of proline and two structure-determining α-amino acids.^10^ Ergotamine and its *8S*-epimer ergotaminine, shown in Figure 1, are primary representatives of the ergopeptines. In 2021, the European Union first established maximum levels for EAs in foodstuffs in Commission Regulation (EU) 2021/1399.^12^ The recently adopted regulation 2023/915 will further lower the maximum values to 400 μg/kg in wheat gluten and 250 μg/kg in rye milling products, down to 20 μg/kg for processed cereal-based food for infants and young children.^13^ The evaluation of contamination levels with EAs in foodstuffs focused on the most abundant EAs (priority EAs) in , which is the main source of EAs in Europe. The maximum levels of EAs are defined as the lower-bound sum of the simple lysergic acid amide ergometrine/ergometrinine and the ergopeptines ergotamine/ergotaminine, ergosine/ergosinine, ergocornine/ergocorninine, ergocristine/ergocristinine, and ergocryptine/ergocryptinine (α- and β-form).^13^ Table 1 provides the specific amino acids, in addition to proline, of the cyclol-lactam ring for the different ergopeptines.

**Table 1:**
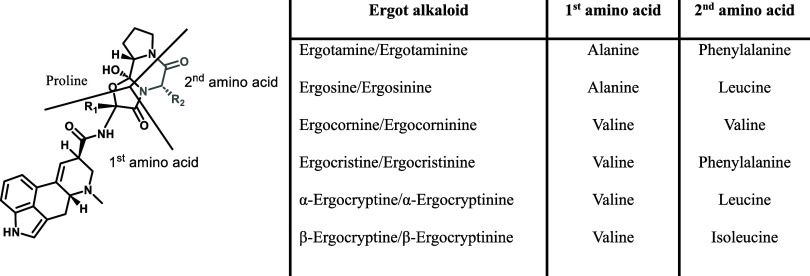
General Structure and Name of the Priority Ergopeptines and the Two Unique Amino Acids of the Cyclol-Lactam Ring

In parallel with the establishment of the maximum levels, the European Standard Method (ESM) EN 17425 for the determination of ergot alkaloids in cereals and cereal-based products was published.^14^ The method describes the extraction of EAs from cereals and the subsequent cleanup of the extract using dispersive solid-phase extraction. The determination of EAs is performed with high-performance liquid chromatography coupled to tandem mass spectrometry (HPLC-MS/MS). However, the European Committee for Standardization (CEN) has indicated that the method cannot be considered satisfactory for levels below 24 μg/kg for the sum of the priority EAs due to an increase in interlaboratory uncertainties observed during the method validation study.^14^ HPLC-MS/MS is susceptible to various forms of interference when used for quantification. A common challenge is the effect of coeluting molecules on the ionization yield of the analyte molecules. These matrix-dependent signal suppression or enhancements impact the analyte signal and cause the measured concentration to be different from its true value.^15,16^ To overcome this problem, matrix-matched calibration can be employed. This involves the use of a calibration solution whose matrix matches the sample matrix, except for the analyte. However, these specific matrices are rarely available in environmental and food analytical studies. Alternatively, an internal standard (ISTD) can be used to reduce the impact of the matrix on the analyte signal. A suitable ISTD has comparable physio-chemical properties to the analyte, e.g., retention time, ionization response, and fragmentation pattern. Because of the need for an appropriate ISTD for each analyte of interest, this approach is costly and time-consuming. Therefore, in HPLC-MS/MS, ISTDs are typically stable isotope-labeled analogs (^2^H, ^13^C, and ^15^N) of the analyte.^17^ For EAs, only ergometrine-^13^CD_3_ and its epimer ergometrinine-^13^CD_3_ are currently commercially available, due to their less complex structure compared to the ergopeptides.^15,18^ However, a comprehensive set of all isotopically labeled priority EAs is required to enhance the current ESM and thus to reliably control the EA maximum level in food for infants and young children at 20 μg/kg. In our previous work, we described a straightforward two-step semisynthesis of the ergopeptines ^13^CD_3_-ergotamine and ^13^CD_3_-ergotaminine, starting from native ergotamine.^19^ The general approach for the two-step semisynthesis of isotopically labeled EAs is illustrated in Figure 2. In the first step, native EA is demethylated at the *N* ^6^-position via an iron-catalyzed dealkylation reaction to yield the corresponding Norergot alkaloid (Nor EA). Subsequent methylation at the *N* ^6^-atom with an isotopically labeled methylating reagent, e.g., iodomethane, yields the isotopically labeled EA.

**Figure 2:**
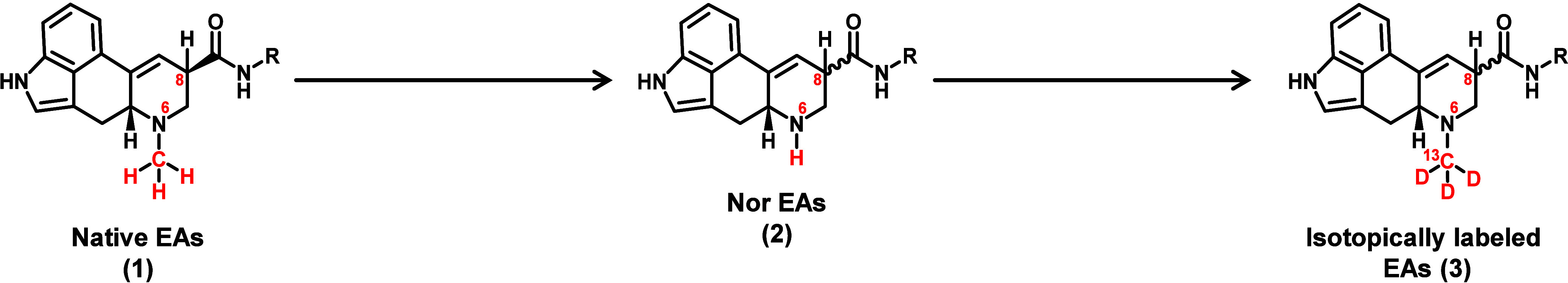
General approach for the two-step synthesis of isotopically labeled EAs. In the first step, native EA (1) is demethylated at the *N* ^6^-atom to yield the corresponding Nor EA (2). Subsequent remethylation with an isotopically labeled methylation reagent yields the isotopically labeled EA (3).

Given that all priority EAs share the methyl group at the *N* ^6^-atom, we investigated the possibility of synthesizing all isotopically labeled EAs via a two-step synthesis.

## Materials and Methods

### Chemicals and Equipment

All chemicals were used without further purification. Ergotamine-D-tartrate, ergometrine-maleate, 3-chloroperoxybenzoic acid (mCPBA, ≤77%), iron powder (for analysis, 10 μm), *N,N*-diisopropylethylamine (≥99%), iron­(III) chloride hexahydrate (≥99%), and ammonium hydroxide solution (25%) were purchased from Sigma-Aldrich (Merck KGaA, Darmstadt, Germany). Ergosine mesylate, ergocornine, ergocristine, and α-ergocryptine had a purity of at least 70% and were bought from ALFARMA s.r.o (Cernosice, Czech Republic). Isotopically labeled iodomethane (^13^CD_3_-I) was purchased from Eurisotop (Saint-Aubin, France). Methanol (MeOH), iso-propanol, dichloromethane, acetone, dimethylformamide, and acetonitrile were all LC-grade or higher and obtained from Th. Geyer (Renningen, Germany).

### Preparative Purification

Measurements for reaction control were carried out on an Agilent 1290 Infinity HPLC system (Agilent, Waldbronn, Germany) with a Phenomenex Gemini-NX C18 (150 × 2.0 mm; 3 μm) column coupled to a 6130 quadrupole MS (Agilent).

Preparative purification was performed on an Agilent LC system consisting of a 1260 Infinity quaternary pump, a 1200 Autosampler, and a column oven coupled to an Agilent 1200 Diode Array Detector with the wavelength set to 310 nm. A Foxy R1 Fraction Collector (Teledyne Isco, Lincoln, NE, USA) was used to automate sample fractionation. Separation was conducted on a Knauer Eurospher II 100–5 C18 P column (250 × 4 mm; 5 μm) or on a Phenomenex Gemini C_6_–Phenyl column (250 × 4.6 mm; 5 μm).

Volatile solvents were removed by nitrogen blowdown in a Reacti-Therm Heating and Stirring Module (Thermo Fischer Scientific, Waltham, MA, USA). Purified products were dried in a rotary vacuum concentrator RVC 2–25 CDplus (Christ, Osterode am Harz, Germany). For thermoshaking, an HLC MHR-13 thermoshaker (Hettich, Tübingen, Germany) was used.

### Chemical- and Isotopic Purity

The chemical purity of each isotope-labeled EA was assessed using an Agilent 1290 Infinity HPLC system equipped with a Phenomenex Gemini C_6_–Phenyl column (150 × 2.0 mm; 3 μm) and coupled to an Agilent Ultivo triple-quadrupole mass spectrometer operating in scan mode (mass-to-charge (*m*/*z*) ratio: 200–900). Isotopic purity, relative to the corresponding native ergot alkaloid, was calculated based on peak areas obtained via multireaction monitoring. The HPLC parameters are provided in the Supporting Information Table S7, while the source- and multireaction monitoring parameters are listed in the Supporting Information Table S8.

### Epimer Ratio

The epimer ratio of the stable isotope-labeled EAs was determined by high-performance liquid chromatography coupled with a fluorescence detector (HPLC-FLD). Therefore, an Agilent LC system consisting of a 1260 Infinity quaternary pump, a 1200 Autosampler, and a column oven coupled to an Agilent 1260 Fluorescence Detector was used. The excitation wavelength was set to 330 nm, the emission wavelength was set to 415 nm, and separation was conducted on a Phenomenex Gemini C_6_–Phenyl column (150 × 3.0 mm; 5 μm). To determine the epimer ratios, aliquots were taken from each epimerically pure stock solution, diluted, and analyzed. The ratios were calculated based on the corresponding peak areas.

### HR-MS/MS Measurements

High-resolution tandem mass spectra (HR-MS/MS) were measured on a Quadrupole time-of-flight 6600 mass spectrometer (Sciex, Darmstadt, Germany). The purified solution, comprising either the native EA or Nor EA, was injected directly into the mass spectrometer via a syringe pump for analysis. For HPLC-HR-MS/MS analysis, the QTOF was coupled to a 1290 Infinity II system (Agilent, Waldbronn, Germany). For the measurements, a Phenomenex Gemini C_6_–Phenyl (150 × 2.0 mm; 3 μm) column was used. For the MS/MS experiments, an inclusion list with the exact *m*/*z* ratio [M + H]^+^ for the native and isotopically labeled EAs was created. Table S6 presents the QTOF parameters, and Table S7 presents the HPLC parameters that were used for the HPLC-HR-MS/MS measurement. The predicted sum formulas for the most abundant product ion *m*/*z* ratios were calculated using a threshold of ±5 ppm deviation (Tables S9–S14).

### Synthesis of Norergot Alkaloids and Isotopically Labeled Ergot Alkaloids

The EA, either as a free base or as a salt, was dissolved or suspended in a suitable solvent (methanol or dichloromethane). The solution or suspension was cooled in an ice bath, and *meta*-chloroperbenzoic acid was added. After complete conversion of the EA, hydrochloric acid, ferric chloride, and iron powder were added to the solution and shaken overnight. The formed demethylated EA was purified by preparative HPLC and dried. The Nor EA was redissolved in acetone, and the remethylation was accomplished using ^13^CD_3_-labeled iodomethane. The crude mixture was purified by preparative HPLC to yield the isotopically labeled EA. A detailed description and the yield of each synthetic step are provided in the Supporting Information for all investigated EAs.

## Results

### Synthesis of Norergot Alkaloids

Previously, we investigated the feasibility of the *N* ^6^-demethylation of ergotamine for the semisynthesis of isotopically labeled ergotamine and ergotaminine. Therein, we achieved *N* ^6^-demethylation to norergotamine via an iron-catalyzed dealkylation reaction. Furthermore, we conducted experiments to assess the impact of different oxidizing agents, iron species, and solvents on the outcome and yield of the reaction.^19^ In this study, we transferred the synthesis parameters from our previous work to address the general unavailability of isotopically labeled EAs. Given their greater availability, the *8R*-configuration of the EAs was utilized as the starting material. Figure 3 depicts the reaction mechanism for the *N* ^6^-demethylation of EAs via an iron-catalyzed dealkylation reaction. The free base of the EA was dissolved in dichloromethane, while ergometrine-maleate and ergometrine-D-tartrate were suspended in methanol due to their higher solubility in a more polar solvent. The addition of mCPBA results in the oxidation of the EA at the *N* ^6^-position, forming the corresponding *N* ^6^-oxide. This result was observed to be consistent across all the investigated EAs. The *N* ^6^-oxide was isolated or directly employed in the subsequent reaction. Without isolation of the *N* ^6^-oxide, hydrochloric acid and Fe^0^ (iron powder) were directly added to the reaction mixture after the oxidation with mCPBA. The elementary iron forms in situ, the catalytically active Fe^2+^ species, where a redox pair of Fe^2+^/Fe^3+^ is believed to sequentially reduce the *N^6^*-oxide to the Nor EA. The primary byproduct of this process is the parent EA, which was recovered and can be reused. Throughout the reaction, epimerization was observed for all of the Nor EAs and the recovered EAs. This phenomenon has been described in the scientific literature and is attributed to various factors, including high or low pH, elevated temperatures, exposure to light, and the presence of protic solvents.^9^ Both *C* ^8^-isomers of the Nor EAs were purified by using preparative HPLC for subsequent reactions. Chromatograms for the preparative purification of individual Nor EAs are given in the Supporting Information Figures S1–S6.

**Figure 3:**
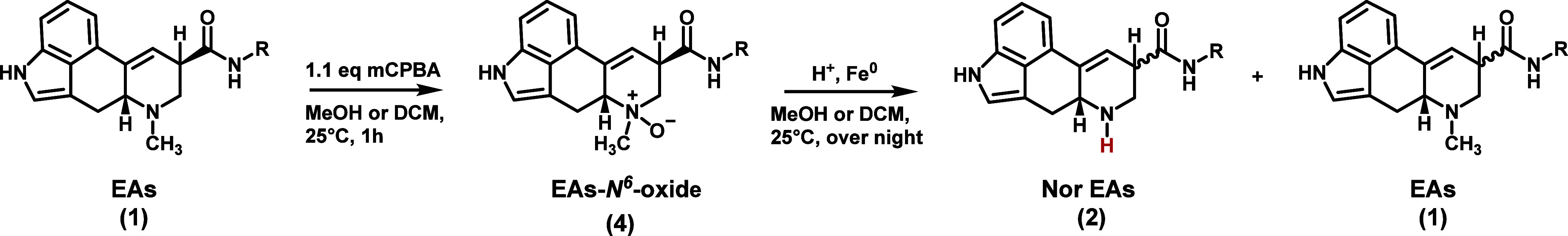
Reaction for the *N* ^6^-demethylation of EAs via an iron-catalyzed N-dealkylation reaction.

### HR-MS/MS of Norergot Alkaloids

The successful *N* ^6^-demethylation of the EAs to the Nor EAs was confirmed by HR-MS/MS. Purified samples containing either the native EA or the associated Nor EA were introduced into the QTOF mass spectrometer for measurement via direct infusion. The HR-MS/MS spectra of the native EAs and Nor EAs were compared, and specific fragment ions were assigned to the major peaks in the mass spectra. Figure 4 shows the comparison of the fragment spectra of native ergocristine and the demethylated product norergocristine. The HR-MS/MS spectra of ergocristine coincide with previously reported data in the literature.^20,21^ Upon comparison of the spectra of ergocristine and norergocristine, a similar fragmentation pattern is evident. The observed differences in the intensities of certain fragment ions can be attributed to the lower energy required for the fragmentation of norergocristine. For fragment ions containing the *N* ^6^-atom, a shift in the *m*/*z* ratio for norergocristine compared to ergocristine was observed. This *m*/*z* shift corresponds to the loss of the methyl group at the *N* ^6^-atom and is observed for the fragment ions of ergocristine/norergocristine with the *m*/*z* ratios of 348.1711/334.1545, 268.1451/254.1280, and 223.1234/209.1073. The differences in the *m*/*z* are 14.0166, 14.0171, and 14.0164, respectively, and match with the *m*/*z* ratio of the neutral loss of CH_2_, which is 14.0157 (cf. Figure 4). The fragmentation of the amide bond between the lysergic acid moiety and the cyclic tripeptide corresponds to the fragment ions with *m*/*z* ratios of 223.1234/209.1070 and 268.1451/254.1280. Fragmentation of the cyclic tripeptide ring results in the fragment ions with *m*/*z* ratios of 348.1711/334.1545. The fragment ion with an *m*/*z* ratio of 325.1546 is indicative of the fragment ion of the tricyclic peptide. Given that no modifications were introduced to the cyclic tripeptide ring structure, the spectra of ergocristine and norergocristine both exhibit this specific fragment ion. The HR-MS/MS spectra for the Nor EAs of ergometrine, ergocornine, ergotamine, α-ergocryptine, and ergosine compared to the native EAs are presented in the Supporting Information, Figures S7, S9, S11, S13, S15. The related structure of all investigated ergopeptines results in a comparable fragmentation pattern, similar to the one observed for ergocristine and norergocristine. In contrast, the representatives of the lysergic acid derivatives, ergometrine and norergometrine, exhibit a less complex product ion spectrum. Based on the measured accurate *m*/*z* ratios, we were able to make a structural proposal for all major product ions of the EAs and Nor EAs.

**Figure 4:**
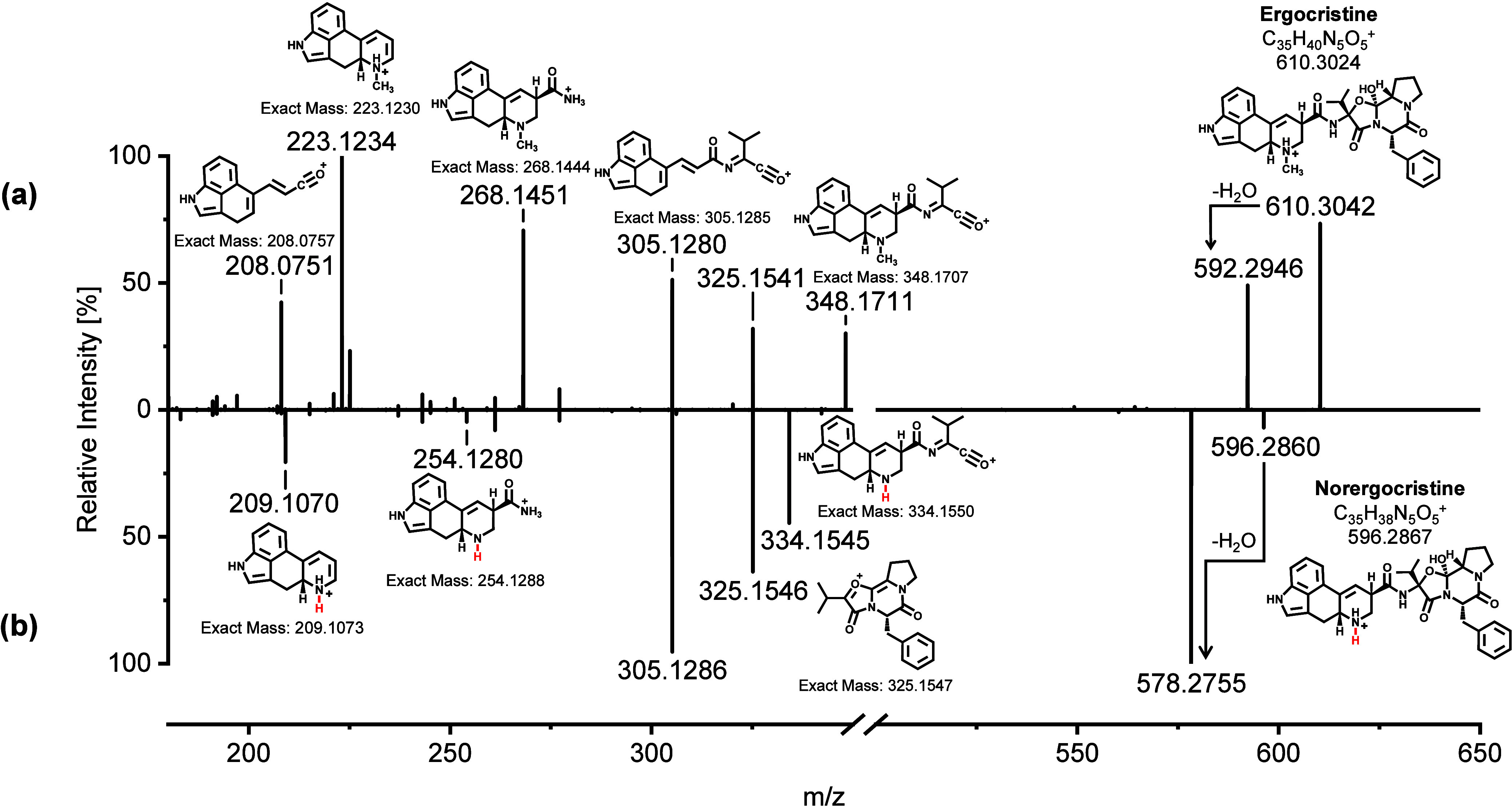
Positive ESI-HR-MS/MS spectra [M + H]^+^ of (a) ergocristine and (b) norergocristine. In addition to the measured accurate *m*/*z* ratio, a suggestive structure is provided for the precursor ion and the major fragment ions along with their calculated exact *m*/*z* ratio.

### Synthesis of Stable Isotope-Labeled Ergot Alkaloids

The remethylation of the Nor EAs was conducted with ^13^CD_3_-iodomethane, and the corresponding isotopically labeled EAs were purified by preparative HPLC to remove any unreacted Nor EA. This procedure yielded the pure and isotopically labeled *C* ^8^-*R* and *C* ^8^-*S* epimers of the corresponding EA. The overall yield for the synthesis of the isotopically labeled EAs and their epimer ratio are given in Table 2.

**Table 2:** Yield and Epimer Ratios for the Synthesis of Stable Isotope-Labeled Priority EAs^a^

**ergot alkaloid-** ^**13**^ **CD**_**3**_	ergometrine & ergometrinine	ergotamine & ergotaminine	ergosine & ergosinine	ergocornine & ergocorninine	ergocryptine & ergocryptinine	ergocristine & ergocristinine
yield [%]	-	14.9	-	19.6	29.5	8.1
epimer ratio [R%:S%]	69:31	51:49	53:47	58:42	51:49	45:55

a Due to the low amount of starting material for ergometrine and ergosine, no yield could be determined for ^13^CD_3_-ergometrine/-inine and ^13^CD_3_-ergosine/-inine.

To verify the successful synthesis of all 12 isotopically labeled priority EAs, they were spiked at a concentration of 25 ng/mL to a standard with a concentration of 25 ng/mL containing all unlabeled native EAs in acetonitrile and analyzed via HPLC-HR-MS/MS. Figure 5 depicts the extracted-ion chromatogram (XIC) for the specific fragment ion of the native (*m*/*z* 223.1235) and isotopically labeled (*m*/*z* 227.1458) EAs.^22^

**Figure 5:**
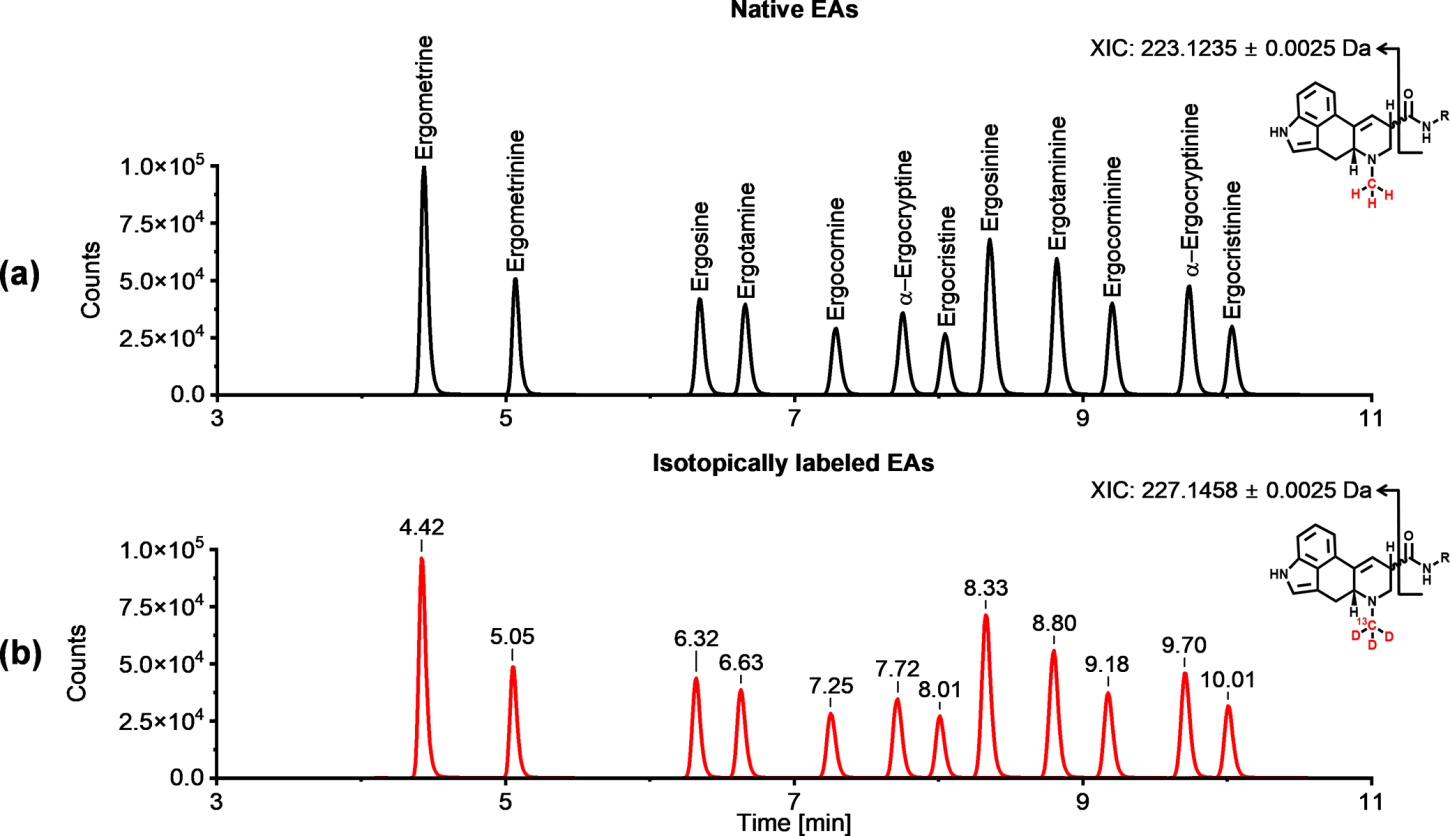
Extracted-ion chromatogram [M + H]^+^ (XIC) of a 25 ng/mL standard for (a) 12 native priority EAs and (b) 12 stable isotope-labeled priority EAs. An inclusion list containing the exact *m*/*z* values of each native and stable isotope-labeled EA [M + H]^+^ was created, and the sample was analyzed in parallel reaction monitoring (PRM) mode on a QTOF mass spectrometer.

The fragment encloses the ergoline structure with the *N* ^6^-atom, resulting in an *m*/*z* shift of 4 Da between the native and isotopically labeled EAs. The retention times of the native and isotopically labeled EAs match; however, a slight shift to earlier retention times can be observed for the isotopically labeled EAs (1–3 s). This phenomenon is predominantly observed for deuterated ISTDs, as the carbon-deuterium bond is more polarized due to the lower electronegativity of deuterium compared to that of hydrogen. Consequently, the isotopically labeled EAs are more polar than the native EAs and elute slightly earlier in reverse-phase HPLC.^23,24^ Figure 6a,b compares the HPLC-HR-MS/MS spectra of ergocristine/^13^CD_3_-ergocristine and its epimer ergocristinine/^13^CD_3_-ergocristinine. The HR-MS/MS spectra of the isotopically labeled EA exhibit a shift in the *m*/*z* ratio of 4 Da for fragment ions that include the isotopically labeled *N* ^6^-atom. Conversely, all other accurate *m*/*z* ratios and intensities were identical to those observed for the native EA. With the measured accurate *m*/*z*, we were able to predict a sum formula and make a structural proposal for the major fragment ions of ^13^CD_3_-ergocristine (Figure 6c). The predicted sum formula, calculated exact *m*/*z* ratio, measured accurate *m*/*z* ratio, and deviation for ^13^CD_3_-ergocristine are presented in Table S9. Due to the analogous structural characteristics of the investigated ergopeptines, they all exhibit a comparable product ion spectrum. As representatives of simple lysergic acid amides, the fragment spectra of native and isotopically labeled ergometrine and ergometrinine differ from those of the ergopeptine group. Nevertheless, distinctive fragment ions are present in both groups, for instance, the cleavage of the alkyl-carbonyl bond between the lysergic acid moiety and the peptide bond, with *m*/*z* ratios of 223.1235 (for unlabeled EAs) and 227.1458 (for isotopically labeled EAs). The data for the remaining isotopically labeled EAs are presented in the Supporting Information Figures S8, S10, S12, S14, and S16 along with the calculated exact *m*/*z* ratio for the specific fragment ion, the measured accurate *m*/*z* ratio, and their derivation (Tables S10–S14).

**Figure 6:**
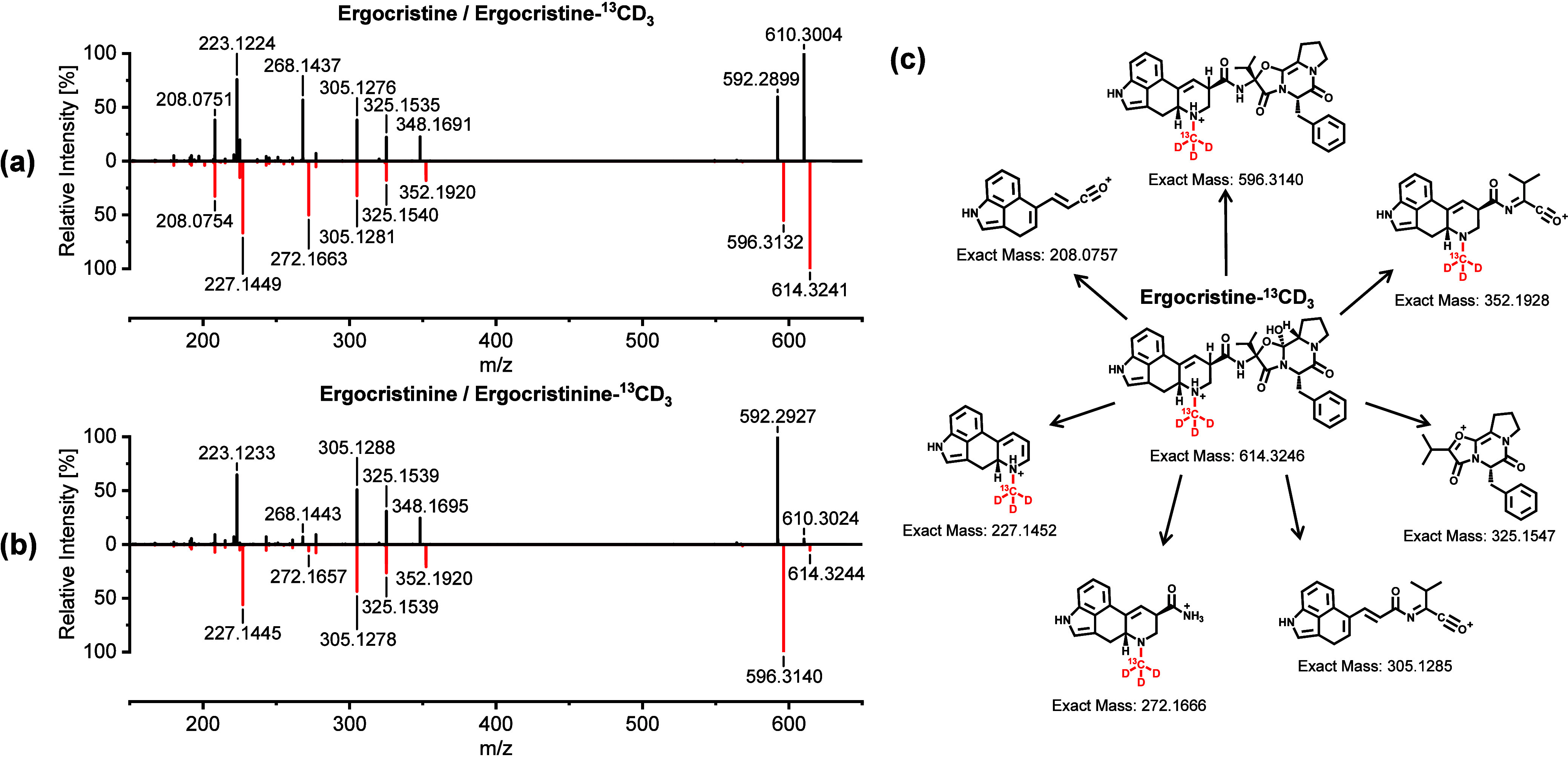
Positive ESI-HR-MS/MS spectra [M + H]^+^ of (a) unlabeled ergocristine (black) and stable isotope-labeled ergocristine-^13^CD_3_ (red); (b) unlabeled ergocristinine (black) and stable isotope-labeled ergocristinine-^13^CD_3_ (red); and (c) structural proposal of major fragment ions for ergocristine-^13^CD_3_.

## Discussion

The quantification of EAs in complex food matrices using HPLC-MS/MS is challenging, primarily due to matrix effects and the lack of stable isotope-labeled standards. To address this gap, a semisynthetic approach was developed that targets the *N* ^6^-atom of the lysergic acid moiety, a structural feature shared among all investigated native priority EAs.

The demethylation of the *N* ^6^-atom to the corresponding Nor EAs was achieved via an iron-catalyzed reaction, and the structure of the Nor EAs was confirmed by HR-MS/MS. A structural assignment for the most intense product ions of the Nor EAs was proposed based on the measured accurate *m*/*z* ratios and correlated with specific HR-MS/MS fragments of the native compounds. The characteristic loss in the *m*/*z* value associated with the demethylation was observed only for fragment ions of the Nor EAs that contain the *N* ^6^-atom, thereby confirming successful and selective demethylation for all investigated EAs.

For the subsequent remethylation of the Nor EAs, ^13^CD_3_-iodomethane was utilized and resulted in the formation of the isotopically labeled *C* ^8^ *-R* and *C* ^8^ *-S* epimers of the respective EAs. Overall yields ranged from 8.1% for ^13^CD_3_-ergocristine/-inine and up to 29.5% for ^13^CD_3_-ergocryptine/-inine. Due to the limited availability of starting material, no yield could be determined for ^13^CD_3_-ergometrine/-inine and ^13^CD_3_-ergosine/-inine. The epimer ratio (R%:S%) of the ergopeptines ranged between 58:42 for ^13^CD_3_-ergocornine/-inine and 45:55 for ^13^CD_3_-ergocristine/-inine. Although the synthesis started from a compound with the *8R*-configuration, both stable isotope-labeled epimers were obtained in approximately equal amounts. This outcome is favorable as it provides sufficient quantities of both stable isotope-labeled epimers from a single epimeric pure starting material. In contrast, the simpler lysergic acid amide ^13^CD_3_-ergometrine/-inine showed less epimerization, with an epimer ratio of 69:31. The structures of the synthesized labeled EAs were confirmed using HPLC-HR-MS/MS. Therefore, a mixture containing all 12 native priority EAs and their corresponding labeled analogs was analyzed. As anticipated for deuterium-labeled compounds, the isotopically labeled EAs showed slightly shorter retention times (1–3 s) compared to their native counterparts. The precursor and fragment ions containing the isotopically labeled methyl group at the *N* ^6^-position exhibited a shift in the *m*/*z* ratio of 4 Da. In contrast, fragment ions without this structural feature were present in the MS/MS spectra of both labeled and unlabeled EAs. With the measured accurate *m*/*z* ratios of the precursor and product ions, we were able to predict a sum formula and match specific fragment ions for each major signal in the HR-MS/MS spectrum. Due to the limited availability of the native EAs, only small amounts of the ^13^CD_3_-labeled analogs were synthesized, which precluded structural elucidation by NMR. However, the identical fragmentation patterns and signal intensities between labeled and unlabeled EAs, in conjunction with the same retention time in HPLC, provided unambiguous confirmation of the structures for all 12 synthesized stable isotope-labeled priority EAs.

For the first time, the procedure described herein enabled the semisynthesis of a complete set of stable isotope-labeled priority EAs. In future work, these standards will be implemented in the ESM and facilitate an enhanced methodology for the quantification of these mycotoxins in food and feed.

## Supplementary Material



## Notes

Detailed synthetic procedure, HR-MS/MS data for all Nor EAs, native, and isotopically labeled EAs (PDF)
