# Supplementary material for: Semisynthesis of Stable Isotope-Labeled Ergot Alkaloids for HPLC-MS/MS Analysis
Source: J Agric Food Chem. 2025 Jul 11;73(29):18412–9. doi: 10.1021/acs.jafc.5c03345 (PMC12291449; doi:10.1021/acs.jafc.5c03345)
Supplement: Supplementary file 1 [file jf5c03345_si_001.pdf]

Supplementary Material

Semi-synthesis of stable isotope-labeled ergot alkaloids for HPLC-MS/MS analysis

Sven-Oliver Herter[a], Hajo Haase[b] and Matthias Koch[a,*]

a Bundesanstalt für Materialforschung und -prüfung (BAM), Division 1.7 Organic Trace and Food Analysis, Richard-Willstätter-Str. 11, 12489 Berlin, Germany

b Technische Universität Berlin, Department of Food Chemistry and Toxicology, Gustav-Meyer-Allee 25, 13355 Berlin, Germany

* E-mail: matthias.koch@bam.de

Contents

1. Synthesis of norergot alkaloids and isotopically labeled ergot alkaloids 3

Synthesis of α-Norergocryptine and α-Norergocryptinine 3

Synthesis of α-Ergocryptine-$^{13}CD_3$ and α-Ergocryptinine-$^{13}CD_3$ 4

Synthesis of Norergocristine and Norergocristinine 5

Synthesis of Ergocristine-$^{13}CD_3$ and Ergocristinine-$^{13}CD_3$ 6

Synthesis of Norergocornine and Norergocorninine 6

Synthesis of Ergocornine-$^{13}CD_3$ and Ergocorninine-$^{13}CD_3$ 7

Synthesis of Norergosine and Norergosinine 7

Synthesis of Ergosine-$^{13}CD_3$ and Ergosinine-$^{13}CD_3$ 8

Synthesis of Norergometrine and Norergometrinine 9

Synthesis of Ergometrine-$^{13}CD_3$ and Ergometrinine-$^{13}CD_3$ 11

Synthesis of Ergotamine-$^{13}CD_3$ and Ergotaminine-$^{13}CD_3$ 11

2. Characterization of norergot alkaloids and stable isotope-labeled ergot alkaloids.... 15

Table of product ions of $^{13}CD_3$-Ergocristine 15

Spectra of Norergometrine and $^{13}CD_3$-Ergometrine/-inine 15

Spectra of Norergosine and $^{13}CD_3$-Ergosine/-inine 17

Spectra of Norergocornine and $^{13}CD_3$-Ergocornine/-inine 19

Spectra of α-Norergocryptine and $^{13}CD_3$-Ergocryptine/-inine 21

Table of Figures

Figure S1. Chromatogram for the purification of norergocryptine and norergocryptinine. 4
Figure S2. Chromatogram for the purification of norergocristine and norergocristinine. 6
Figure S3. Chromatogram for the purification of norergocornine and norergocorninine. 7
Figure S4. Chromatogram for the purification of norergosine and norergosinine. 8
Figure S5. Chromatogram for the purification of norergometrine and norergometrinine. 10
Figure S6. Chromatogram for the purification of norergotamine and norergotaminine. 12
Figure S7. HR-ESI-MS/MS of ergometrine and norergometrine. 15
Figure S8. HR-ESI-MS/MS spectra of native ergometrine/-inine and isotopically labeled ergometrine/-inine. 16
Figure S9. HR-ESI-MS/MS of ergosine and norergosinine. 17
Figure S10. HR-ESI-MS/MS spectra of native ergosine/-inine and isotopically labeled ergosine/-inine 17
Figure S11. HR-ESI-MS/MS of ergocornine and norergocornine. 19
Figure S12. HR-ESI-MS/MS spectra of native ergocornine/-inine and isotopically labeled ergocornine/-inine 19
Figure S13. HR-ESI-MS/MS of α-ergocryptine and α-norergocryptine. 21
Figure S14. HR-ESI-MS/MS spectra of native α-ergocryptine/-inine and isotopically labeled α-ergocryptine/-inine 22
Figure S15. HR-ESI-MS/MS of ergotamine and norergotamine. 23
Figure S16. HR-ESI-MS/MS spectra of native ergotamine/-inine and isotopically labeled ergotamine/-inine 24

1. Synthesis of norergot alkaloids and isotopically labeled ergot alkaloids

Synthesis of α-Norergocryptine and α-Norergocryptinine

α-Ergocryptine (11.1 mg, 19.3 µmol, 1.0 eq.) was dissolved in 2 mL dichloromethane and cooled down in an ice bath. After 15 min mCPBA (4.75 mg, 21.2 µmol, 1.1 eq) was added to the ice-cooled solution and the reaction was stirred for 1 hour at room temperature or until no α-ergocryptine was detected by HPLC-MS. The magnetic stir bar was removed and 1 molar hydrochloric acid in methanol (38.6 µL, 2.0 eq.), 5 g/L $FeCl_3\,6{\times}H_2O$ (52.2 µL, 0.05 eq.) and iron powder (10.4 mg, 186.2 µmol, 9.6 eq) were added to the solution and shaken for 3 hours at 35 °C. The complete conversion of the α-ergocryptine-N^6-oxide was confirmed by HPLC-MS. The color of the reaction changed from light yellow at the beginning to deep red-brown at the end. After the reaction was finished, the solvent was removed by blowdown evaporation and the residue was redissolved in 1 mL acetonitrile/water (70 v% / 30 v%). The solution was basified with a solution of 0.5 molar trisodium phosphate in water (96.5 µL, 2.5 eq). The precipitate was removed by centrifugation and the supernatant was purified by preparative HPLC (Table S1, Figure S1) to yield 3.9 mg (6.9 µmol; 35.7 %) of α-norergocryptine and α-norergocryptinine.

m/z (measured) $(M+H)^+$ = 562.3036; theoretical $(M+H)^+$: 562.3024 , δ = 2.1 ppm

Table S1. Preparative LC conditions: Phenomenex Gemini C_6-Phenyl (250 × 4.6 mm; 5 µm) column; flow rate: 1 mL/min, column oven temperature: 35 °C; injection volume: 100 µL; runtime: 31 min; eluents: H_2O + 2 mM NH_3; acetonitrile; DAD wavelength: 310 nm.

Time [min]	H_2O + 2 mM NH_3 [%]	Acetonitrile [%]
0	66	34
20	66	34
20.1	0	100
25	0	100
25.1	66	34
31	66	34

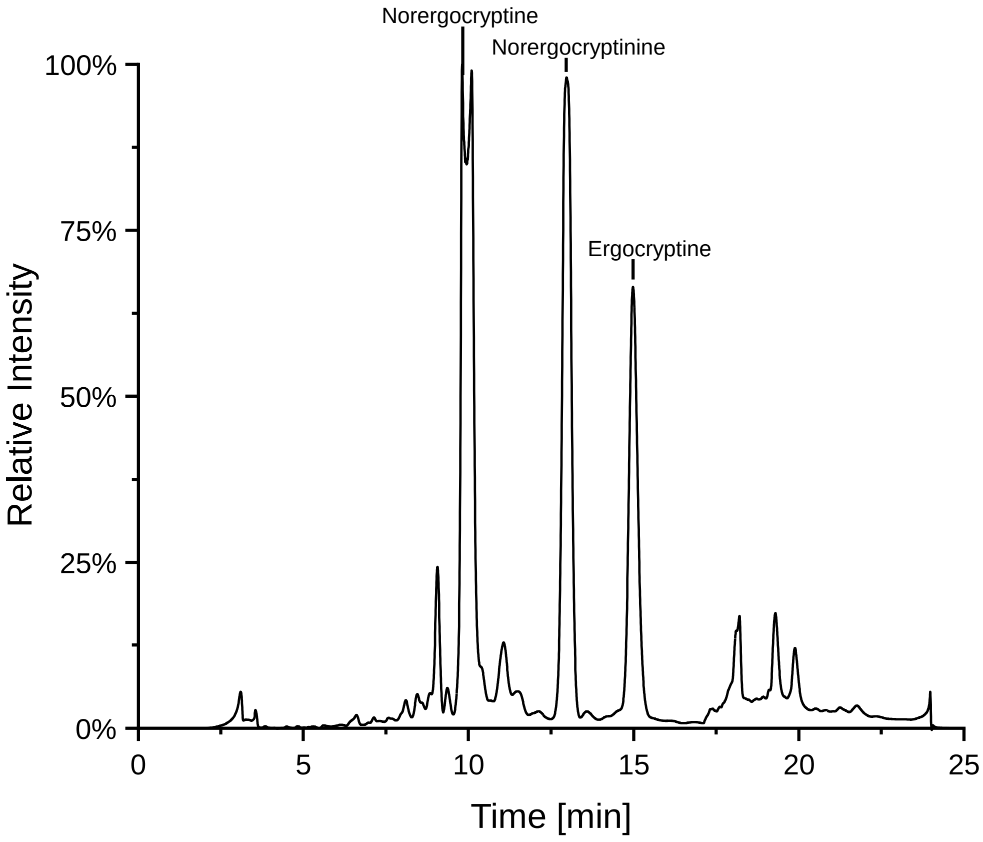

Figure S1. Preparative HPLC-DAD (λ=310 nm) chromatogram for the purification of norergocryptine and norergocryptinine.

Synthesis of α-Ergocryptine-$^{13}CD_3$ and α-Ergocryptinine-$^{13}CD_3$

A mixture of α-norergocryptine and α-norergocryptinine (3.9 mg, 6.9 µmol, 1.0 eq.) was dissolved in 500 µL acetone. *N,N*-Diisopropylethylamine (10.4 µmol, 1.5 eq.) and $^{13}CD_3$-I (10.4 µmol, 1.5 eq) were added to the solution and shaken at room temperature for 24 hours. The solvent was removed in a rotary vacuum concentrator and the residue was redissolved in 300 µL acetonitrile / water+20 mM NH_3 (80 v % / 20 v %). The crude mixture was purified via preparative HPLC (Table S2**Error! Reference source not found.**) to yield 3.3 mg (5.7 µmol, 82.6 %) α-ergocryptine-$^{13}CD_3$ and α-ergocryptinine-$^{13}CD_3$ with an epimer ratio (R %:S %) of 51:49 and a chemical and isotopic purity >99 %.

Table S2. Preparative LC conditions: Phenomenex Gemini C_6-Phenyl (250 × 4.6 mm; 5 µm) column; flow rate: 1 mL/min; column oven temperature: 35 °C; injection volume: 100 µL; runtime: 33 min; eluents: H_2O + 2 mM NH_3; acetonitrile; DAD wavelength: 310 nm.

**Time [min]**	**H_2O + 2 mM NH_3 [%]**	**Acetonitrile [%]**
0	70	30
10	55	45
16	40	60
22	30	70
22.1	0	100
27	0	100
27.1	70	30
33	70	30

m/z (measured) $(M+H)^+$ = 580.3394; theoretical $(M+H)^+$: 580.3402, δ = -1.4 ppm

Synthesis of Norergocristine and Norergocristinine

Ergocristine (10.3 mg, 16.9 µmol, 1.0 eq.) was dissolved in 2 mL dichloromethane and cooled down in an ice bath. After 15 min mCPBA (4.16 mg, 18.6 µmol, 1.1 eq) was added to the ice-cooled solution and the reaction was stirred for 1 hour at room temperature or until no ergocristine was detected by HPLC-MS. The magnetic stir bar was removed and 1 molar hydrochloric acid in methanol (33.8 µL, 2.0 eq.), 5 g/L $FeCl_3$ 6×H_2O (46.6 µL, 0.05 eq.) and iron powder (9.6 mg, 171.9 µmol, 10.2 eq) were added to the solution and shaken for 3 hours at 35 °C The complete conversion of the ergocristine-N^6-oxide was confirmed by HPLC-MS. The color of the reaction changed from light yellow at the beginning to deep red-brown at the end. After the reaction was finished, the solvent was removed by blowdown evaporation and the residue was redissolved in 1 mL acetonitrile/water (70 v% / 30 v%). The solution was basified with a solution of 0.5 molar trisodium phosphate in water (84.5 µL, 2.5 eq). The precipitate was removed by centrifugation and the supernatant was purified by preparative HPLC (Table S1, Figure S2) to yield 1.8 mg (3.0 µmol, 10.6 %) of norergocristine and norergocristinine.

m/z (measured) $(M+H)^+$ = 596.2860; theoretical $(M+H)^+$: 596.2867, δ = -1.2 ppm

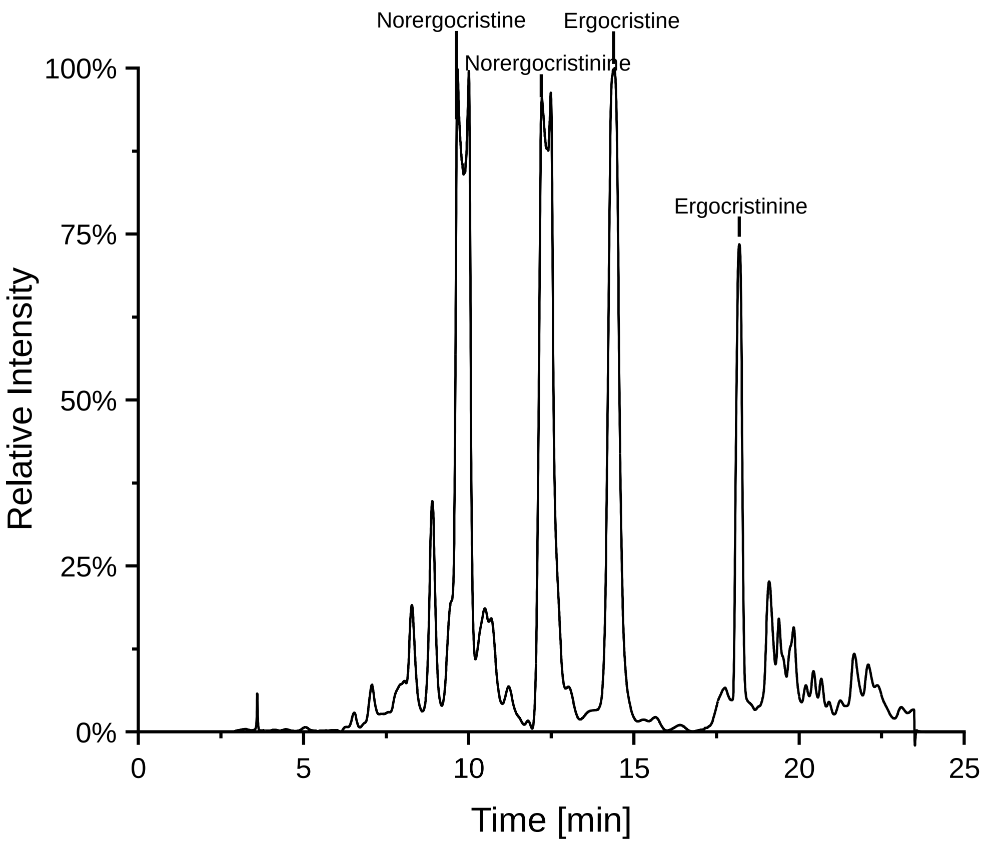

Figure S2. Preparative HPLC-DAD (λ=310 nm) chromatogram for the purification of norergocristine and norergocristinine.

Synthesis of Ergocristine-$^{13}CD_3$ and Ergocristinine-$^{13}CD_3$

A mixture of norergocristine and norergocristinine (1.8 mg, 3.0 µmol, 1.0 eq.) was dissolved in 300 µL acetone. *N,N*-Diisopropylethylamine (4.5 µmol, 1.5 eq.) and $^{13}CD_3$-I (4.5 µmol, 1.5 eq) were added to the solution and shaken at room temperature for 24 hours. The solvent was removed in a rotary vacuum concentrator and the residue was redissolved in 200 µL acetonitrile / water+20 mM NH_3 (80 v % / 20 v %). The crude mixture was purified via preparative HPLC (Table S2) to yield 1.4 mg (2.3 µmol, 76.6 %) ergocristine-$^{13}CD_3$ and ergocristinine-$^{13}CD_3$ with an epimer ratio (R %:S %) of 45:55 and a chemical and isotopic purity >99 %.

m/z (measured) $(M+H)^+$ = 614.3246; theoretical $(M+H)^+$: 614.3241, δ = -0.8 ppm

Synthesis of Norergocornine and Norergocorninine

Ergocornine (10.0 mg, 17.8 µmol, 1.0 eq.) was dissolved in 2 mL dichloromethane and cooled down in an ice bath. After 15 min mCPBA (4.38 mg, 19.6 µmol, 1.1 eq) was added to the ice-cooled solution and the reaction was stirred for 1 hour at room temperature or until no ergocornine was detected by HPLC-MS. The magnetic stir bar was removed and 1 molar hydrochloric acid in methanol (35.6 µL, 2.0 eq.), 5 g/L $FeCl_3$ 6×H_2O (48.1 µL, 0.05 eq.) and iron powder (10.1 mg, 179.1 µmol, 10.1 eq) were added to the solution and shaken for 3 hours at 35 °C. The complete conversion of the ergocornine-N^6-oxide was confirmed by HPLC-MS. The color of the reaction changed from light yellow at the beginning to deep red-brown at the end. After the reaction was finished, the solvent was removed by blowdown evaporation and the residue

was redissolved in 1 mL acetonitrile/water (70 v% / 30 v%). The solution was basified with a solution of 0.5 molar trisodium phosphate in water (89 µL, 2.5 eq). The precipitate was removed by centrifugation and the supernatant was purified by preparative HPLC (Table S1, Figure S3) to yield 2.4 mg (4.4 µmol, 24.7 %) of norergocornine and norergocorninine.

m/z (measured) $(M+H)^+$ = 548.2870; theoretical $(M+H)^+$: 548.2868, δ = 0.4 ppm

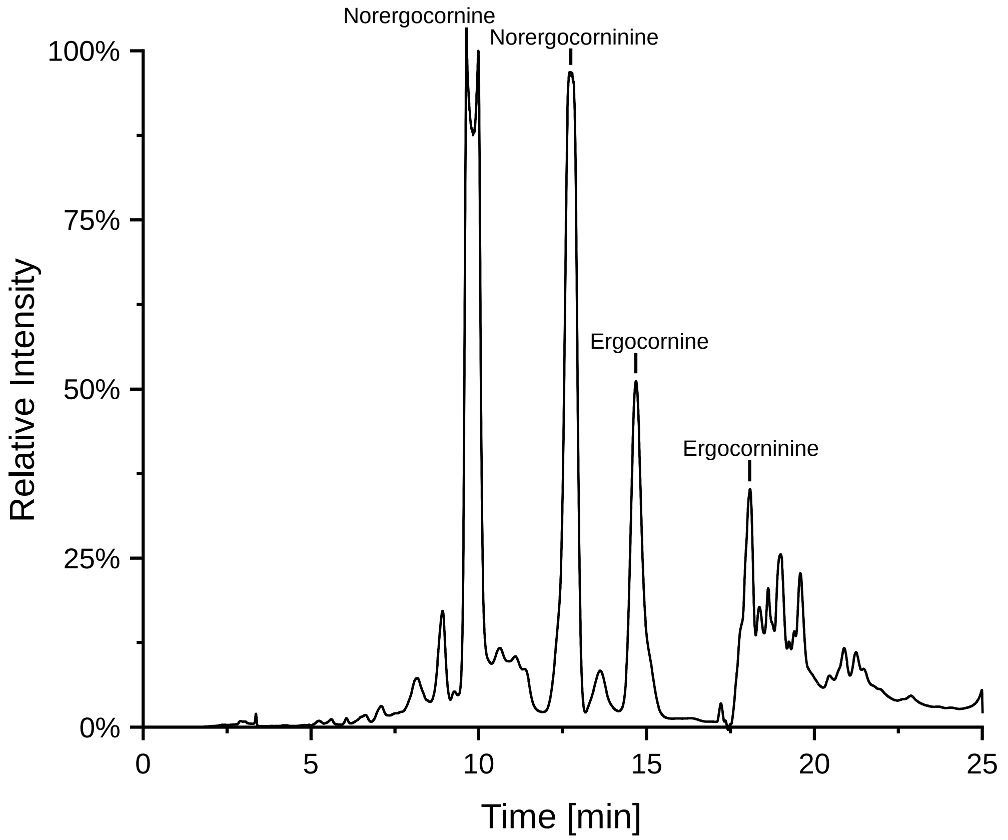

Figure S3. Preparative HPLC-DAD (λ=310 nm) chromatogram for the purification of norergocornine and norergocorninine.

Synthesis of Ergocornine-$^{13}CD_3$ and Ergocorninine-$^{13}CD_3$

A mixture of norergocornine and norergocorninine (2.4 mg, 4.4 µmol, 1.0 eq.) was dissolved in 400 µL acetone. *N,N*-Diisopropylethylamine (6.6 µmol, 1.5 eq.) and $^{13}CD_3$-I (6.6 µmol, 1.5 eq) were added to the solution and shaken at room temperature for 24 hours. The solvent was removed in a rotary vacuum concentrator and the residue was redissolved in 250 µL acetonitrile / water+20 mM NH_3 (80 v % / 20 v %). The crude mixture was purified via preparative HPLC (Table S2) to yield in total 2.0 mg (3.5 µmol, 79.5 %) ergocornine-$^{13}CD_3$ and ergocorninine-$^{13}CD_3$ with an epimer ratio (R %:S %) of 58:42 and a chemical and isotopic purity >98 %.

m/z (measured) $(M+H)^+$ = 566.3240; theoretical $(M+H)^+$: 566.3246, δ = -1.1 ppm

Synthesis of Norergosine and Norergosinine

Ergosine mesylate (5.0 mg, 7.8 µmol, 1.0 eq.) was suspended in 1 mL dichloromethane and 1 molar ammonia solution (8.6 µL, 8.6 µmol, 1.1 eq.) was added to the solution. The solution was vigorously shaken,

and the solid particles dissolved, creating a homogeneous solution. After 10 minutes, the solvent was removed using a rotary vacuum concentrator. The residue was then redissolved in 1 mL of dichloromethane and centrifuged for 3 minutes at 14500 rpm. The resulting supernatant, which contained ergosine as a free base, was used for the subsequent reaction. The solution was cooled down in an ice bath and after 15 min mCPBA (1.74 mg, 8.6 µmol, 1.1 eq) was added to the ice-cooled solution and the reaction was stirred for 1 hour at room temperature or until no ergosine was detected by HPLC-MS. The magnetic stir bar was removed and 1 molar hydrochloric acid in methanol (15.6 µL, 2.0 eq.), 5 g/L $FeCl_3$ 6×H_2O (21.1 µL, 0.05 eq.) and iron powder (4.7 mg, 84.1 µmol, 10.8 eq) were added to the solution and shaken for 3 hours at 35 °C. The complete conversion of the ergosine-N^6-oxide was confirmed by HPLC-MS. The color of the reaction changed from light yellow at the beginning to deep red-brown at the end. After the reaction was finished, the solvent was removed by blowdown evaporation and the residue was redissolved in 500 µL acetonitrile/water (70 v% / 30 v%). The solution was basified with a solution of 0.5 molar trisodium phosphate in water (39 µL, 2.5 eq). The precipitate was removed by centrifugation and the supernatant was purified by preparative HPLC (Table S1, Figure S4) to yield norergosine and norergosinine.

m/z (measured) $(M+H)^+$ = 534.2710; theoretical $(M+H)^+$: 534.2711, δ = -0.2 ppm

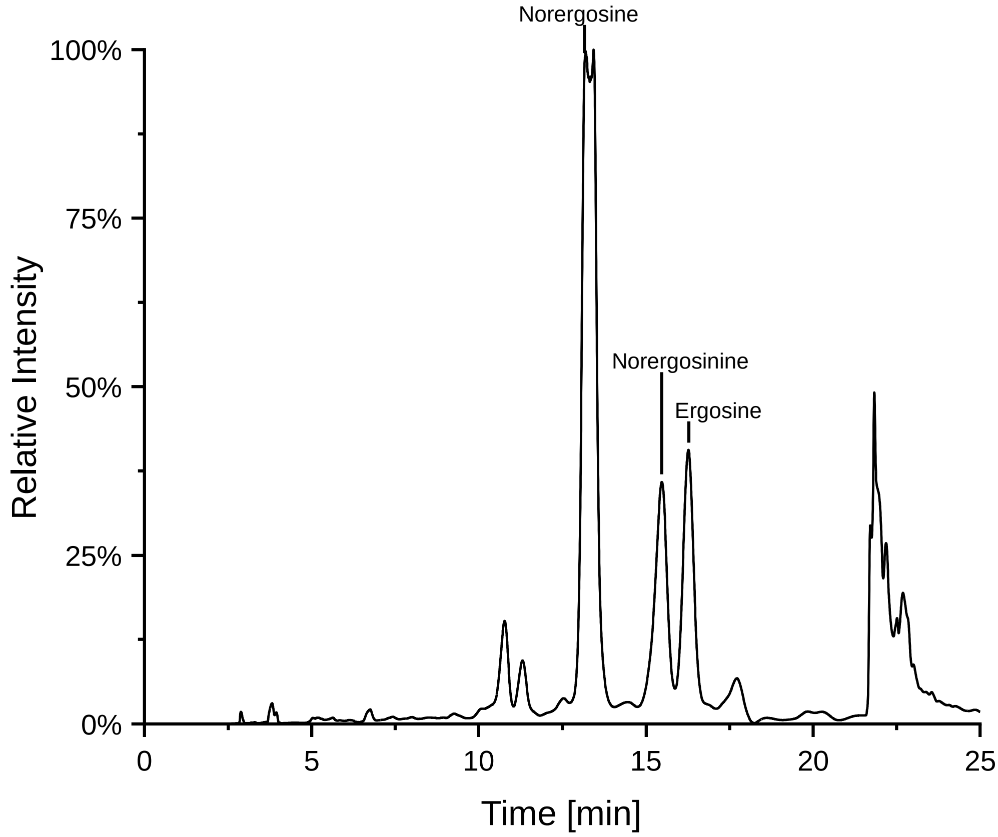

Figure S4. Preparative HPLC-DAD (λ=310 nm) chromatogram for the purification of norergosine and norergosinine.

Synthesis of Ergosine-$^{13}CD_3$ and Ergosinine-$^{13}CD_3$

A mixture of norergosine and norergosinine was dissolved in 200 µL acetone. *N,N*-Diisopropylethylamine (2 µmol,) was added to the solution and subsequently small portions of $^{13}CD_3$-I

in acetone (0.5 µmol/aliquote) was added to the solution and shaken at room temperature. After 1 hour the progress of the reaction was monitored with HPLC-MS and if substantial amounts of norergosine/-inine was detected, another aliquot was added. When only a minor amount of norergosine/-inine was detected with HPLC-MS, the solvent was removed in a rotary vacuum concentrator and the residue was redissolved in 250 µL acetonitrile / water + 20 mM NH_3 (80 v % / 20 v %). The crude mixture was purified via preparative HPLC (Table S2) to yield ergosine-$^{13}CD_3$ and ergosinine-$^{13}CD_3$ in solution with an epimer ratio (R %:S %) of 53:47 and a chemical and isotopic purity >99 %.

m/z (measured) $(M+H)^+$ = 552.3083 (theoretical $(M+H)^+$: 552.3089, δ = -1.1 ppm

Synthesis of Norergometrine and Norergometrinine

Ergometrine maleate (10.0 mg, 22.7 µmol, 1 eq) was dissolved in 2 mL methanol and cooled down in an ice bath. After 15 min mCPBA (5.6 mg, 25.0 µmol, 1.1 eq) was added to the ice-cooled solution and the reaction was stirred for 1 hour at room temperature or until no ergometrine was detected by HPLC-MS. The magnetic stir bar was removed and 1 molar hydrochloric acid in methanol (45.4 µL, 2.0 eq.), 5 g/L $FeCl_3$ 6×H_2O (61.4 µL, 0.05 eq.) and iron powder (13.1 mg, 234.8 µmol, 10.3 eq) were added to the solution and shaken overnight and the absences of the ergometrine-N^6-oxide was confirmed by HPLC-MS. The color of the reaction changed from light yellow at the beginning to deep red-brown at the end. After the reaction was finished, the solvent was removed by blowdown evaporation and the residue was redissolved in 1 mL acetonitrile/water (50 v% / 50 v%). The solution was basified with a solution of 0.5 molar trisodium phosphate in water (113.5 µL, 2.5 eq). The precipitate was removed by centrifugation and the supernatant was purified by preparative HPLC (Table S3, Figure S5) to yield 1.3 mg (4.2 µmol, 18.5 %) of norergometrine and norergometrinine.

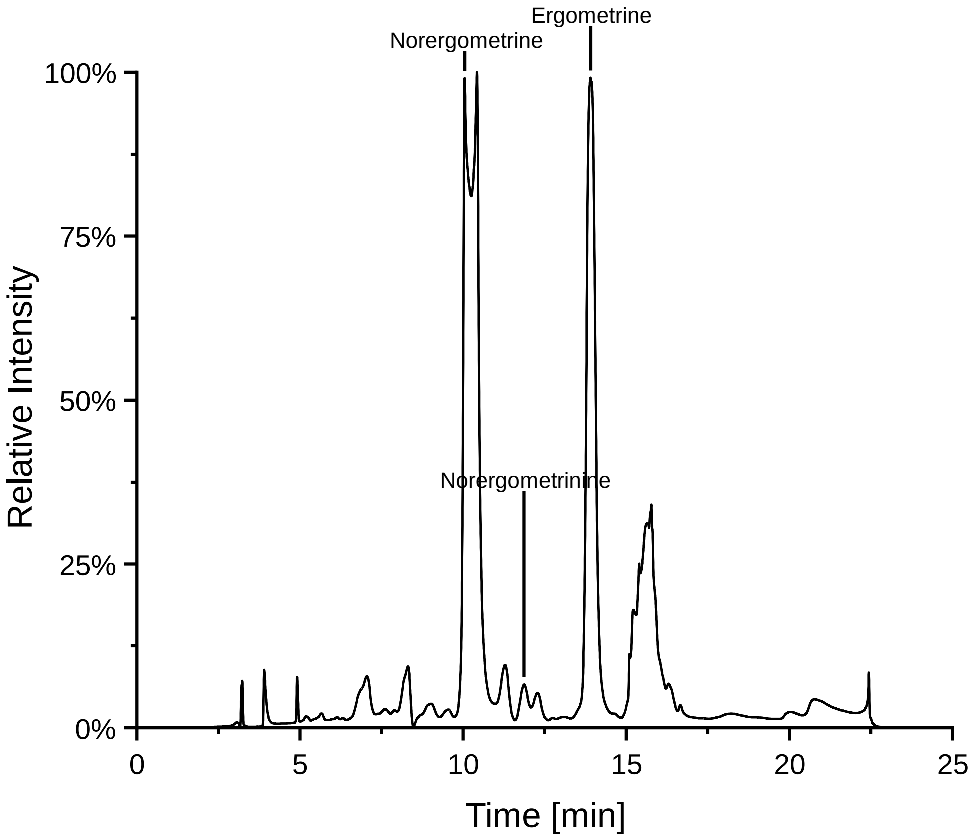

Figure S5. Preparative HPLC-DAD (λ=310 nm) chromatogram for the purification of norergometrine and norergometrinine.

Table S3. Preparative LC conditions: Knauer Eurospher II 100-5 C18 P (250 × 4 mm; 5 µm) column; flow rate: 1 mL/min, column oven temperature: 35 °C; injection volume: 100 µL; runtime: 33 min; eluents: H_2O + 2 mM NH_3; acetonitrile; DAD wavelength: 310 nm.

**Time [min]**	**H_2O + 2 mM NH_3 [%]**	**Acetonitrile [%]**
0	92	8
2	92	8
20	70	30
20.1	0	100
26	0	100
26.1	92	8
33	92	8

m/z (measured) $(M+H)^+$ = 312.1708; theoretical $(M+H)^+$: 312.1707, δ = 0.3 ppm

Synthesis of Ergometrine-$^{13}CD_3$ and Ergometrinine-$^{13}CD_3$

A mixture of norergometrine and norergometrinine (1.3 mg, 4.2 µmol) was dissolved in 200 µL acetone/dimethylformamide (50 v% / 50v %). *N,N*-Diisopropylethylamine (6.3 µmol, 1.5 eq.) and $^{13}CD_3$-I (6.3 µmol, 1.5 eq) were added to the solution and shaken at room temperature for 24 hours. The acetone was removed in a rotary vacuum concentrator and the residue was redissolved in 400 µL water+20 mM NH_3. The crude mixture was purified via preparative HPLC (Table S4) to yield ergometrine-$^{13}CD_3$ and ergometrinine-$^{13}CD_3$ in solution with an epimer ratio (R %:S %) of 69:31 and a chemical and isotopic purity >99 %.

Table S4. Preparative LC conditions: Knauer Eurospher II 100-5 C18 P (250 × 4 mm; 5 µm) column; flow rate: 1 mL/min, column oven temperature: 35 °C; injection volume: 100 µL; runtime: 30 min; eluents: H_2O + 2 mM NH_3; acetonitrile; DAD wavelength: 310 nm.

Time [min]	H_2O + 2 mM NH_3 [%]	Acetonitrile [%]
0	90	10
1.0	90	10
20	50	50
20.1	0	100
25	0	100
25.1	90	10
30	90	10

m/z (measured) $(M+H)^+$ = 330.2082; theoretical $(M+H)^+$: 330.2085, δ = -0.9 ppm

Synthesis of Ergotamine-$^{13}CD_3$ and Ergotaminine-$^{13}CD_3$

Isotopically labeled ergotamine and ergotaminine were synthesized in accordance with our previously described procedure. In brief: ergotamine-d-tartrate was suspended in methanol and cooled down in an ice bath and mCPBA was added to the cooled solution and stirred for 1 hour at room temperature. The magnetic stir bar was removed and hydrochloric acid, iron trichloride and iron powder were added and shaken at 35 °C for 24 hours. The complete conversion of ergotamine-N^6-oxide was confirmed by HPLC-MS. Preparative HPLC yielded the norergotamine and norergotaminine (Table S1, Figure S6). Subsequent remethylation with isotopically labeled iodomethane yielded the isotopically labeled ergotamine-$^{13}CD_3$ and ergotaminine-$^{13}CD_3$ in a total yield of 14.9 % with an epimer ratio (R %:S %) of 51:49 and a chemical and isotopic purity >99 %

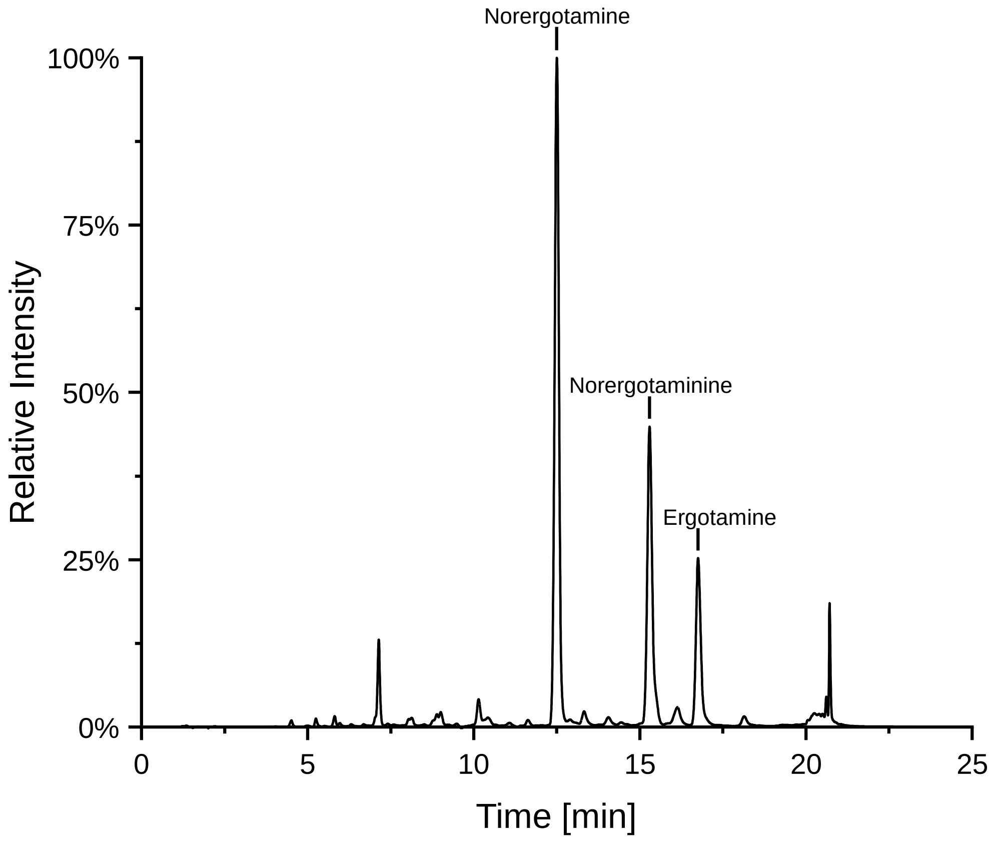

Figure S6. Preparative HPLC-DAD (λ=310 nm) chromatogram for the purification of norergotamine and norergotaminine.

Table S5. Preparative LC conditions: Knauer Eurospher II 100-5 C18 P (250 × 4 mm; 5 µm) column; flow rate: 1 mL/min, column oven temperature: 35 °C; injection volume: 100 µL; runtime: 31 min; eluents: H_2O + 2 mM NH_3; acetonitrile; DAD wavelength: 310 nm.

**Time [min]**	**H_2O + 2 mM NH_3 [%]**	**Acetonitrile [%]**
0	66	34
20	66	34
20.1	0	100
25	0	100
25.1	66	34
31	66	34

Table S6. Parameters for the QTOF 6600 positive ESI-HR-MS/MS measurements.

Parameter—ESI Source		Parameter—Mass Spectrometer	
Temperature	300 °C	MS 1	
Ion Source Gas 1	60 psi	Collision energy	5 V
Ion Source Gas 2	70 psi	Declustering Potential	80 V
Curtain Gas	25 psi	Mass range	300–800
Ionspray Voltage	5500 V	MS 2	
		Collision Energy	40 V
		Collision Energy Spread	20 V
		Declustering Potential	80 V
		Mass range	100–800

Table S7. HPLC conditions: Phenomenex Gemini C_6-Phenyl (150 × 2.0 mm; 3 µm) column; flow rate: 0.4 mL/min; column oven temperature: 40 °C; injection volume: 5 µL; runtime: 16 min; eluents: H_2O + 2 mM NH_3; acetonitrile.

Time [min]	H_2O + 2 mM NH_3 [%]	Acetonitrile [%]
0	95	5
0.5	95	5
3.0	55	45
6.0	45	55
11.0	20	80
11.1	0	100
13.0	0	100
13.1	95	5
16.0	95	5

Table S8. Source parameters of the Agilent Ultivo triple-quadrupole mass spectrometer and the used MRM transitions to assess isotopic purity of the isotopically labeled ergot alkaloids relative to the native ergot alkaloids.

Gas Flow	11 L/min	Gas Temperature	250°C
Nebulizer Pressure	25 psi	Capillary Voltage	2500 V
Sheath Gas Flow	10 L/min	Sheath Gas Temperature	375°C

**Ergot Alkaloid**	**Precursor ion-$^{13}CD_3$ (nativ) $[M+H]^+$**	**Quantifier (nativ)<br>Qualifier 1 (nativ)<br>Qualifier 2 (nativ)**	**Collision Energy [V]**
Ergometrine-$^{13}CD_3$	330.2 (326.2)	227 (223)<br>283 (283)<br>201 (197)	24<br>20<br>24
Ergometrinine-$^{13}CD_3$	330.2 (326.2)	227 (223)<br>283 (283)<br>180 (180)	24<br>20<br>44
Ergosine-$^{13}CD_3$	552.3 (548.3)	227 (223)<br>277 (277)<br>272 (268)	36<br>24<br>24
Ergotamine-$^{13}CD_3$	586.3 (582.3)	227 (223)<br>277 (277)<br>272 (268)	32<br>24<br>20
Ergocornine-$^{13}CD_3$	566.3 (562.3)	227 (223)<br>305 (305)<br>272 (268)	36<br>24<br>24
Ergocryptine-$^{13}CD_3$	580.3 (576.3)	227 (223)<br>305 (305)<br>272 (268)	36<br>24<br>24
Ergocristine-$^{13}CD_3$	614.3 (610.3)	227 (223)<br>305 (305)<br>272 (268)	36<br>28<br>24
Ergosinine-$^{13}CD_3$	552.3 (548.3)	227 (223)<br>277 (277)<br>263 (263)	32<br>24<br>24
Ergotaminine-$^{13}CD_3$	586.3 (582.3)	227 (223)<br>297 (297)<br>277 (277)	32<br>28<br>28
Ergocorninine-$^{13}CD_3$	566.3 (562.3)	227 (223)<br>305 (305)<br>277 (277)	36<br>24<br>28
Ergocryptinine-$^{13}CD_3$	580.3 (576.3)	227 (223)<br>305 (305)<br>291 (291)	36<br>28<br>28
Ergocristinine-$^{13}CD_3$	614.3 (610.3)	227 (223)<br>305 (305)<br>325 (325)	36<br>24<br>24

2. Characterization of norergot alkaloids and stable isotope-labeled ergot alkaloids

Table of product ions of $^{13}CD_3$-Ergocristine

Table S9. Formula, calculated exact *m/z* ratio, measured accurate *m/z* ratio and deviation Δm/m [ppm] for the main product ions $[M+H]^+$ of ergocristine-$^{13}CD_3$.

Formula	Exact *m/z* [M+H]+	Accurate *m/z* [M+H]+	Δm/m [ppm]
$^{13}CC_{34}H_{36}D_3N_5O_5^+$	614.3246	614.3241	-0.8
$^{13}CC_{34}H_{34}D_3N_5O_4^+$	596.3140	596.3132	-1.3
$^{13}CC_{20}H_{18}D_3N_3O_2^+$	352.1919	352.1920	0.3
$C_{19}H_{20}N_2O_3^+$	325.1547	325.1540	-2.2
$C_{19}H_{16}N_2O_2^+$	305.1285	305.1281	-1.3
$^{13}CC_{15}H_{15}D_3N_3O^+$	272.1666	272.1664	-0.7
$^{13}CC_{14}H_{12}D_3N_2^+$	227.1452	227.1449	-1.3
$C_{14}H_{10}NO^+$	208.0757	208.0754	-1.4

Spectra of Norergometrine and $^{13}CD_3$-Ergometrine/-inine

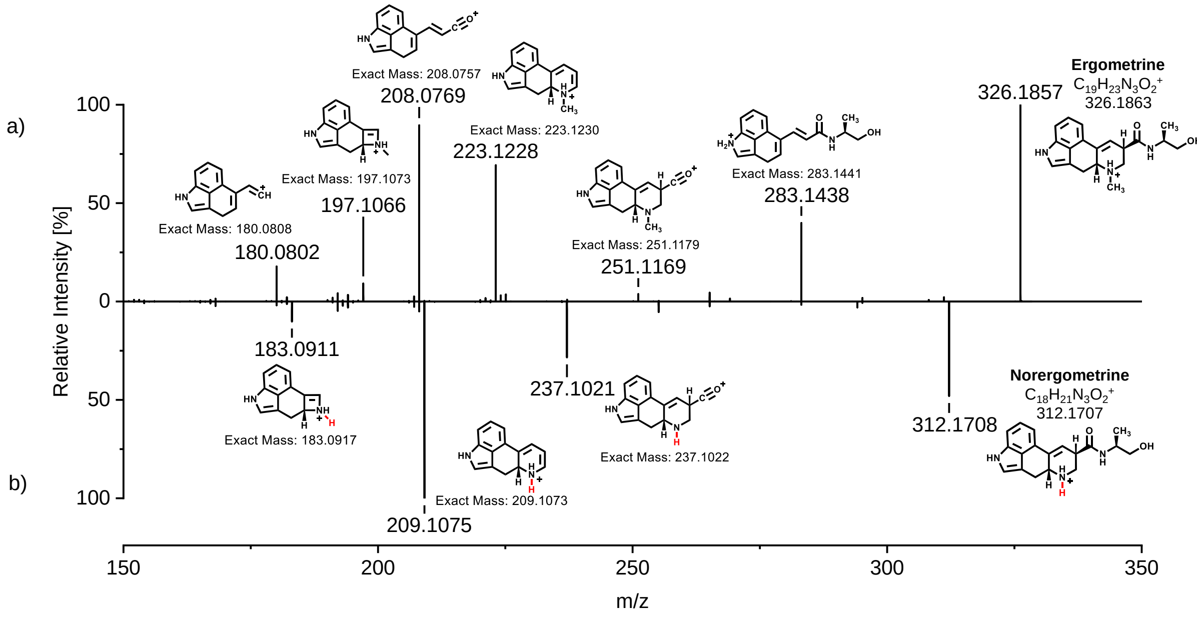

Figure S7. Positive electrospray ionization high-resolution tandem mass spectra $[M+H]^+$ of (a) ergometrine and (b) norergometrine. In addition to the measured accurate *m/z*, a structure is provided for the precursor ion and the major product ions along with their calculated exact *m/z*.

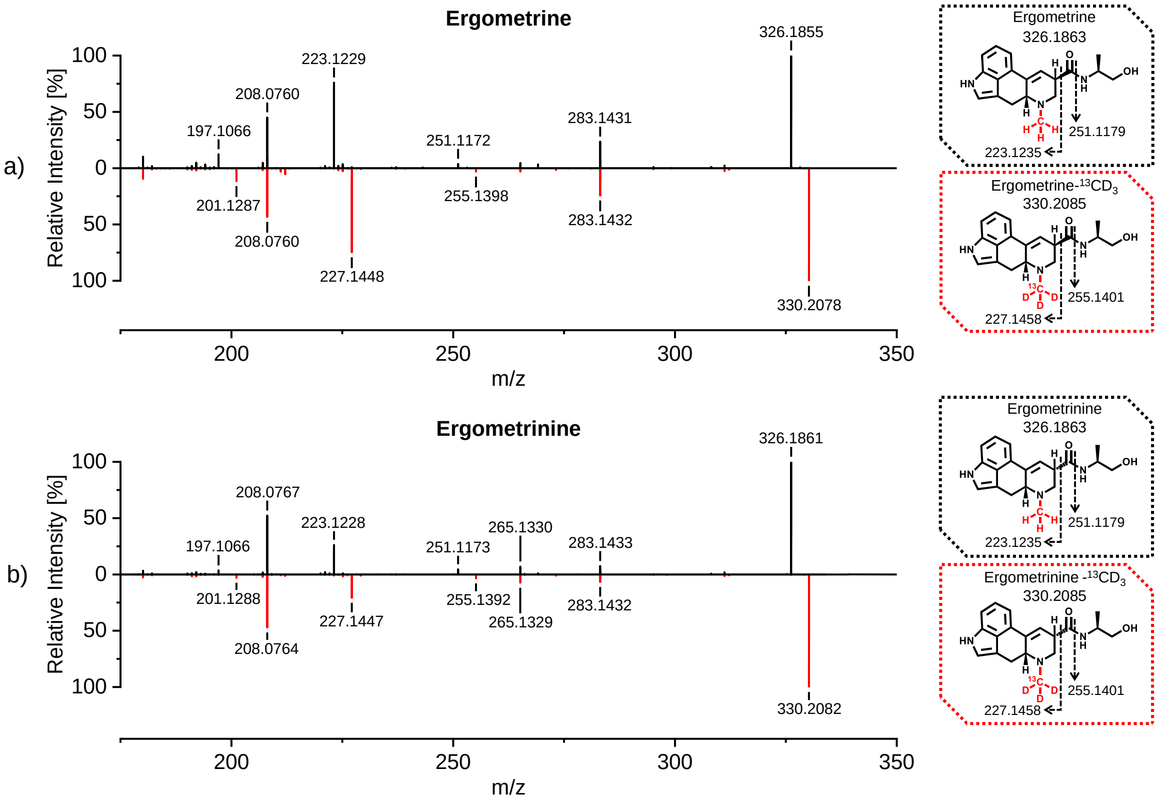

Figure S8. High resolution tandem mass spectra $[M+H]^+$ of a) native ergometrine (black) and isotopically labelled ergometrine-$^{13}CD_3$ (red) and b) native ergometrinine (black) and isotopically labeled ergometrinine-$^{13}CD_3$.

Table S10. Formula, calculated exact *m/z* ratio, measured accurate *m/z* ratio and deviation Δm/m [ppm] for the main product ions $[M+H]^+$ of ergometrinine-$^{13}CD_3$.

Formula	Exact *m/z* $[M+H]^+$	Accurate *m/z* $[M+H]^+$	Δm/m [ppm]
$^{13}CC_{18}H_{20}D_3N_3O_2^+$	330.2085	330.2082	-0.9
$C_{17}H_{18}N_2O_2^+$	283.1441	283.1432	-2.8
$C_{17}H_{16}N_2O^+$	265.1335	265.1329	-2.3
$^{13}CC_{15}H_{11}D_3N_2O^+$	255.1401	255.1392	-3.5
$^{13}CC_{14}H_{12}D_3N_2^+$	227.1458	227.1447	-4.8
$C_{14}H_{10}NO^+$	208.0757	208.0764	3.4
$^{13}CC_{12}H_9D_3N_2^+$	201.1295	201.1288	-3.5

Spectra of Norergosine and $^{13}CD_3$-Ergosine/-inine

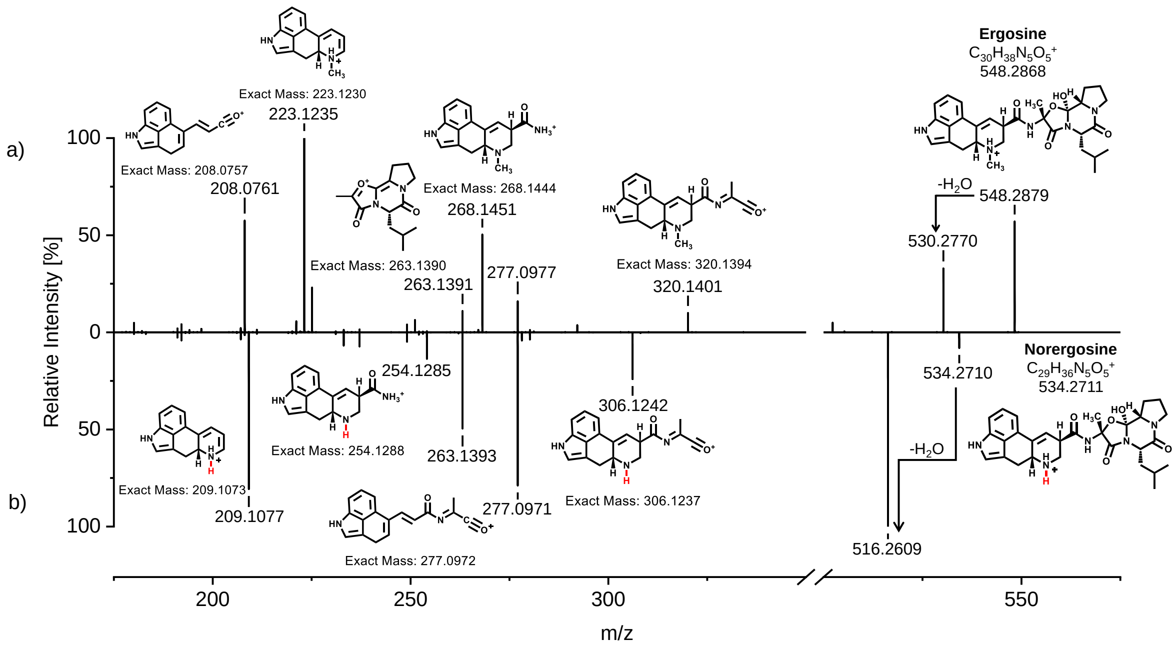

Figure S9. Positive electrospray ionization high-resolution tandem mass spectra [M+H]⁺ of (a) ergosine and (b) norergosine. In addition to the measured accurate *m/z*, a structure is provided for the precursor ion and the major product ions along with their calculated exact *m/z*.

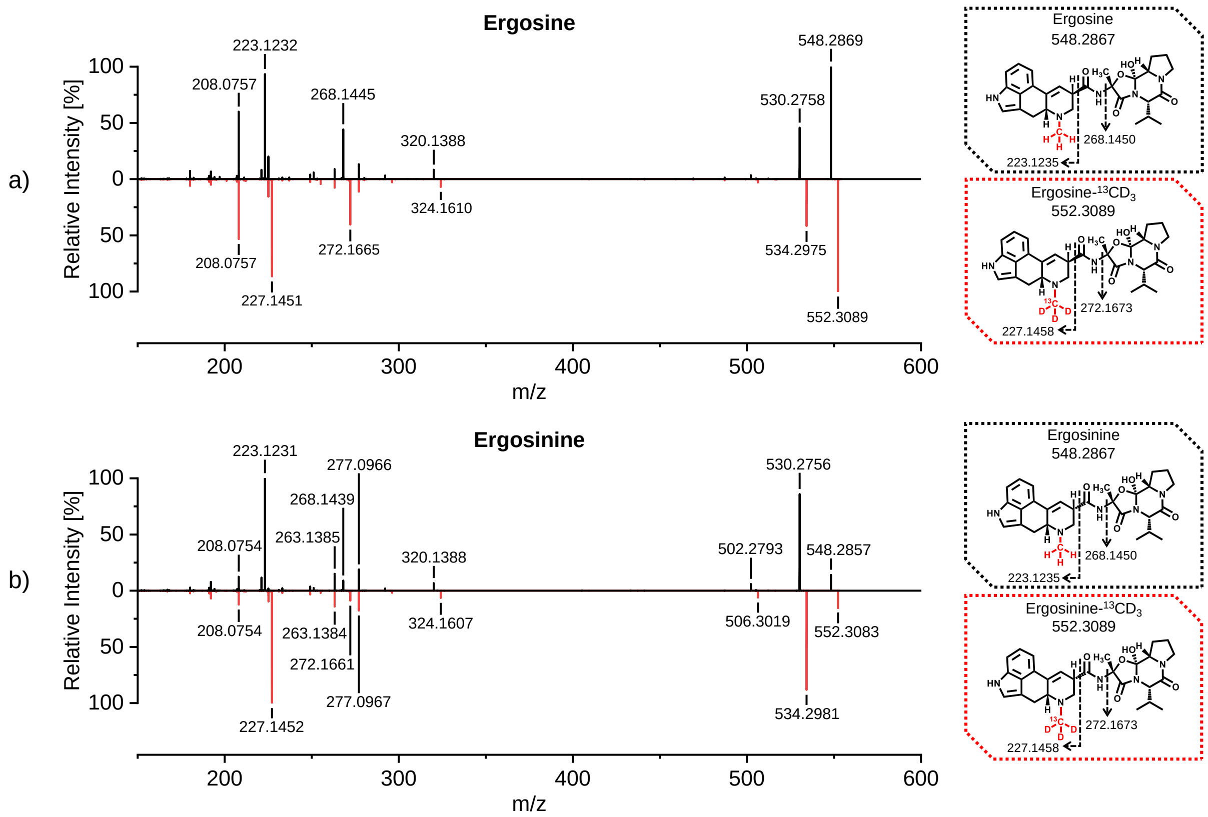

Figure S10. High resolution tandem mass spectra [M+H]⁺ of a) native ergosine (black) and isotopically labelled ergosine-$^{13}CD_3$ (red) and b) native ergosinine (black) and isotopically labeled ergosinine-$^{13}CD_3$.

Table S11. Formula, calculated exact *m/z* ratio, measured accurate *m/z* ratio and deviation Δm/m [ppm] for the main product ions $[M+H]^+$ of ergosinine-$^{13}CD_3$.

Formula	Exact *m/z* $[M+H]^+$	Accurate *m/z* $[M+H]^+$	Δm/m [ppm]
$^{13}CC_{29}H_{34}D_3N_5O_5^+$	552.3089	552.3083	-1.1
$^{13}CC_{29}H_{32}D_3N_5O_4^+$	534.2984	534.2981	-0.6
$^{13}CC_{28}H_{32}D_3N_5O_3^+$	506.3035	506.3019	-3.2
$^{13}CC_{18}H_{14}D_3N_3O_2^+$	324.1615	324.1607	-2.5
$C_{17}H_{12}N_2O_2^+$	277.0972	277.0967	-1.8
$^{13}CC_{15}H_{15}D_3N_3O^+$	272.1666	272.1661	-1.8
$C_{14}H_{18}N_2O_3^+$	263.1390	263.1384	-2.3
$^{13}CC_{14}H_{12}D_3N_2^+$	227.1452	227.1452	0.0
$C_{14}H_{10}NO^+$	208.0757	208.0754	-1.4

Spectra of Norergocornine and $^{13}CD_3$-Ergocornine/-inine

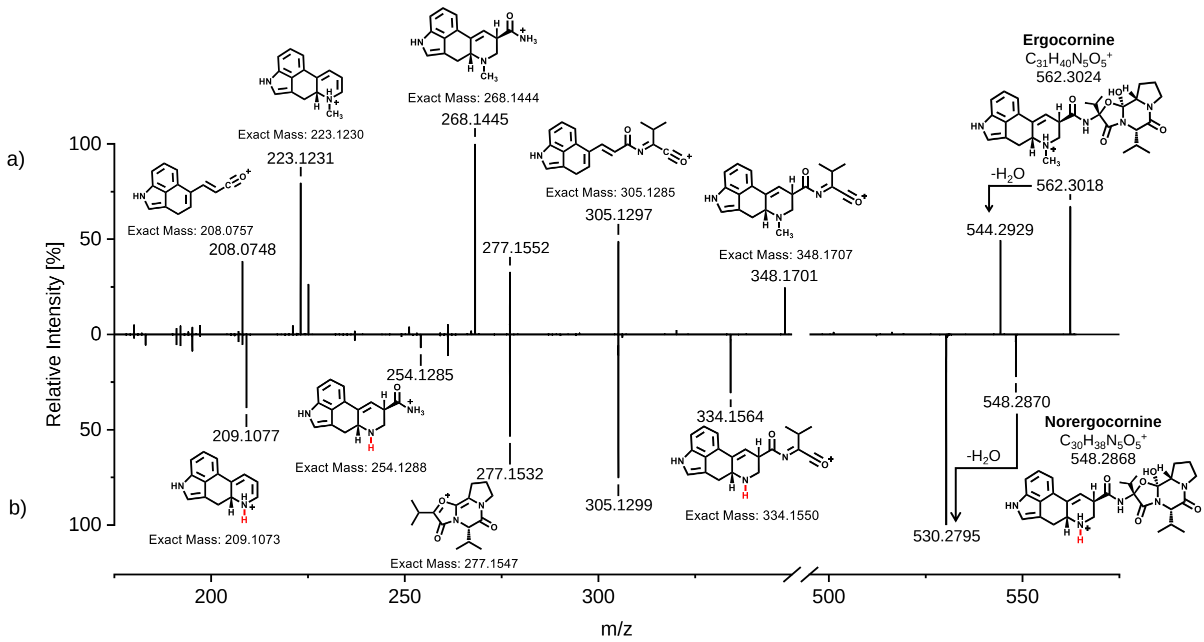

Figure S11. Positive electrospray ionization high-resolution tandem mass spectra [M+H]+ of (a) ergocornine and (b) norergocornine. In addition to the measured accurate *m/z*, a structure is provided for the precursor ion and the major product ions along with their calculated exact *m/z*.

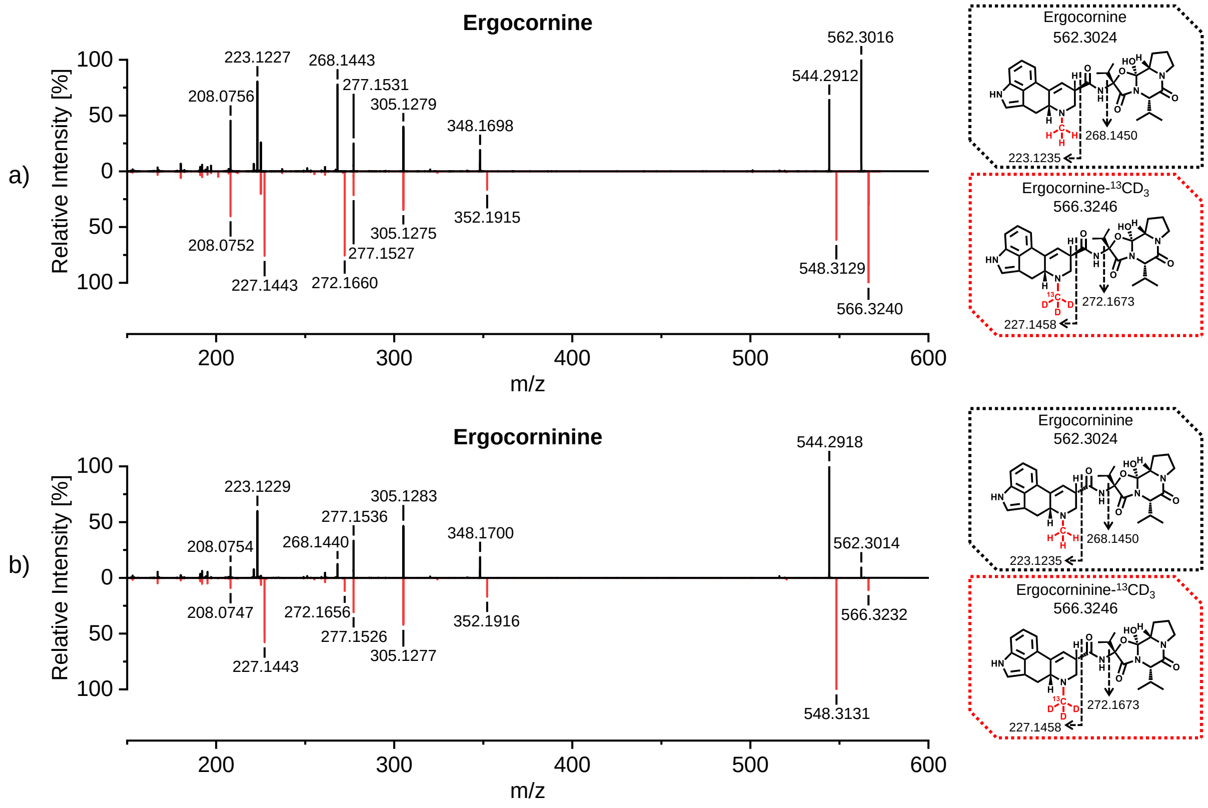

Figure S12. High resolution tandem mass spectra [M+H]+ of a) native ergocornine (black) and isotopically labelled ergocornine-$^{13}CD_3$ (red) and b) native ergocorninine (black) and isotopically labeled ergocorninine-$^{13}CD_3$.

Table S12. Formula, calculated exact *m/z* ratio, measured accurate *m/z* ratio and deviation Δm/m [ppm] for the main product ions $[M+H]^+$ of ergocornine-$^{13}CD_3$.

**Formula**	**Exact *m/z* [M+H]⁺**	**Accurate *m/z* [M+H]⁺**	**Δm/m [ppm]**
$^{13}CC_{30}H_{36}D_3N_5O_5^+$	566.3246	566.3240	-1.1
$^{13}CC_{30}H_{34}D_3N_5O_4^+$	548.3140	548.3129	-2.0
$^{13}CC_{20}H_{18}D_3N_3O_2^+$	352.1919	352.1915	-1.1
$C_{19}H_{16}N_2O_2^+$	305.1285	305.1275	-3.3
$C_{15}H_{20}N_2O_3^+$	277.1547	277.1527	-7.2
$^{13}CC_{15}H_{15}D_3N_3O^+$	272.1666	272.1660	-2.2
$^{13}CC_{14}H_{12}D_3N_2^+$	227.1452	227.1443	-4.0
$C_{14}H_{10}NO^+$	208.0757	208.0752	-2.4

Spectra of α-Norergocryptine and $^{13}CD_3$-Ergocryptine/-inine

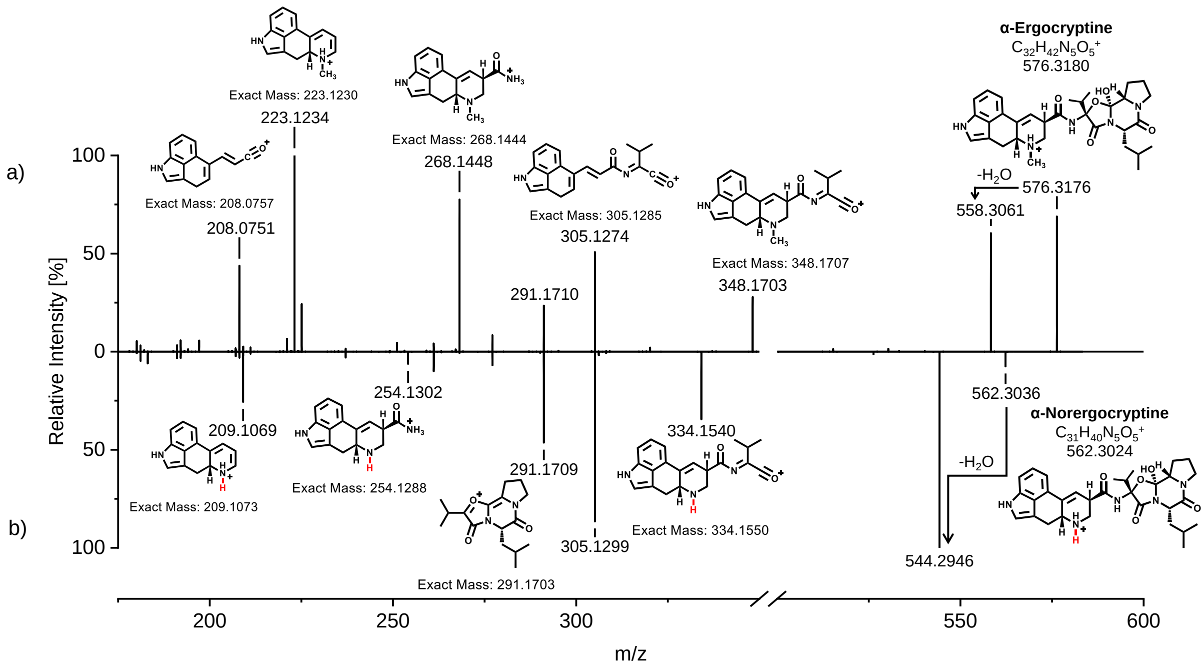

Figure S13. Positive electrospray ionization high-resolution tandem mass spectra [M+H]+ of (a) α-ergocryptine and (b) α-norergocryptine. In addition to the measured accurate *m/z*, a structure is provided for the precursor ion and the major product ions along with their calculated exact *m/z*.

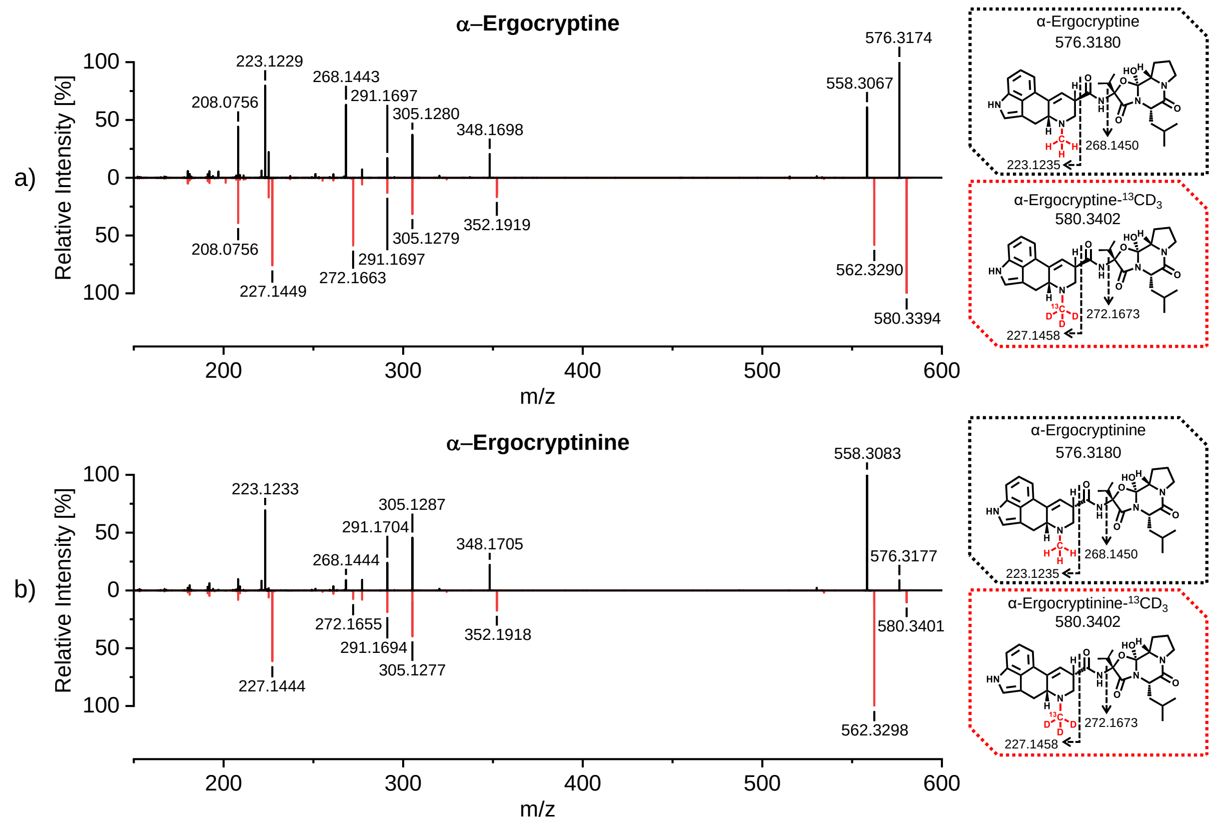

Figure S14. High resolution tandem mass spectra $[M+H]^+$ of a) native α-ergocryptine (black) and isotopically labelled α-ergocryptine-$^{13}CD_3$ (red) and b) native α-ergocryptinine (black) and isotopically labeled α-ergocryptinine-$^{13}CD_3$.

Table S13. Formula, calculated exact *m/z* ratio, measured accurate *m/z* ratio and deviation Δm/m [ppm] for the main product ions $[M+H]^+$ of α-ergocryptine-$^{13}CD_3$.

Formula	Exact *m/z* [M+H]⁺	Accurate *m/z* [M+H]⁺	Δm/m [ppm]
$^{13}CC_{31}H_{38}D_3N_5O_5^+$	580.3402	580.3394	-1.4
$^{13}CC_{31}H_{36}D_3N_5O_4^+$	562.3297	562.3290	-1.2
$^{13}CC_{20}H_{18}D_3N_3O_2^+$	352.1919	352.1919	0.0
$C_{19}H_{16}N_2O_2^+$	305.1285	305.1279	-2.0
$C_{16}H_{22}N_2O_3$	291.1703	291.1697	-2.1
$^{13}CC_{15}H_{15}D_3N_3O^+$	272.1666	272.1663	-1.1
$^{13}CC_{14}H_{12}D_3N_2^+$	227.1452	227.1449	-1.3
$C_{14}H_{10}NO^+$	208.0757	208.0756	-0.5

Spectra of Norergotamine and 13CD3-Ergotamine/-inine

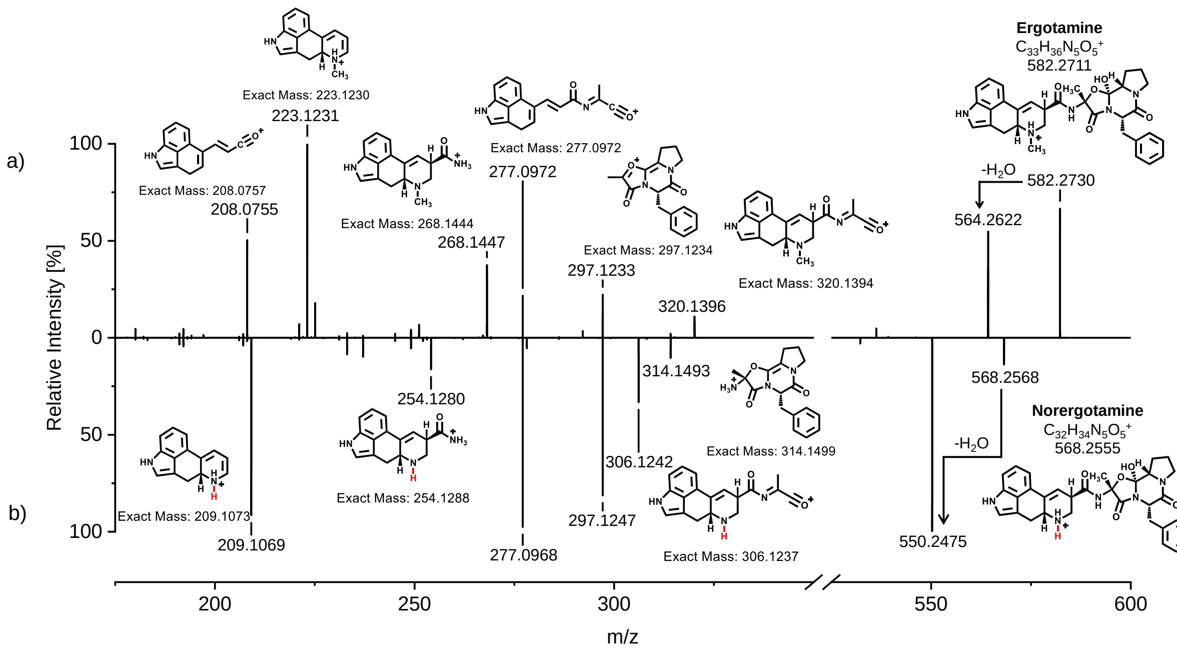

Figure S15. Positive electrospray ionization high-resolution tandem mass spectra $[M+H]^+$ of (a) ergotamine and (b) norergotamine. In addition to the measured accurate *m/z*, a structure is provided for the precursor ion and the major product ions along with their calculated exact *m/z*.

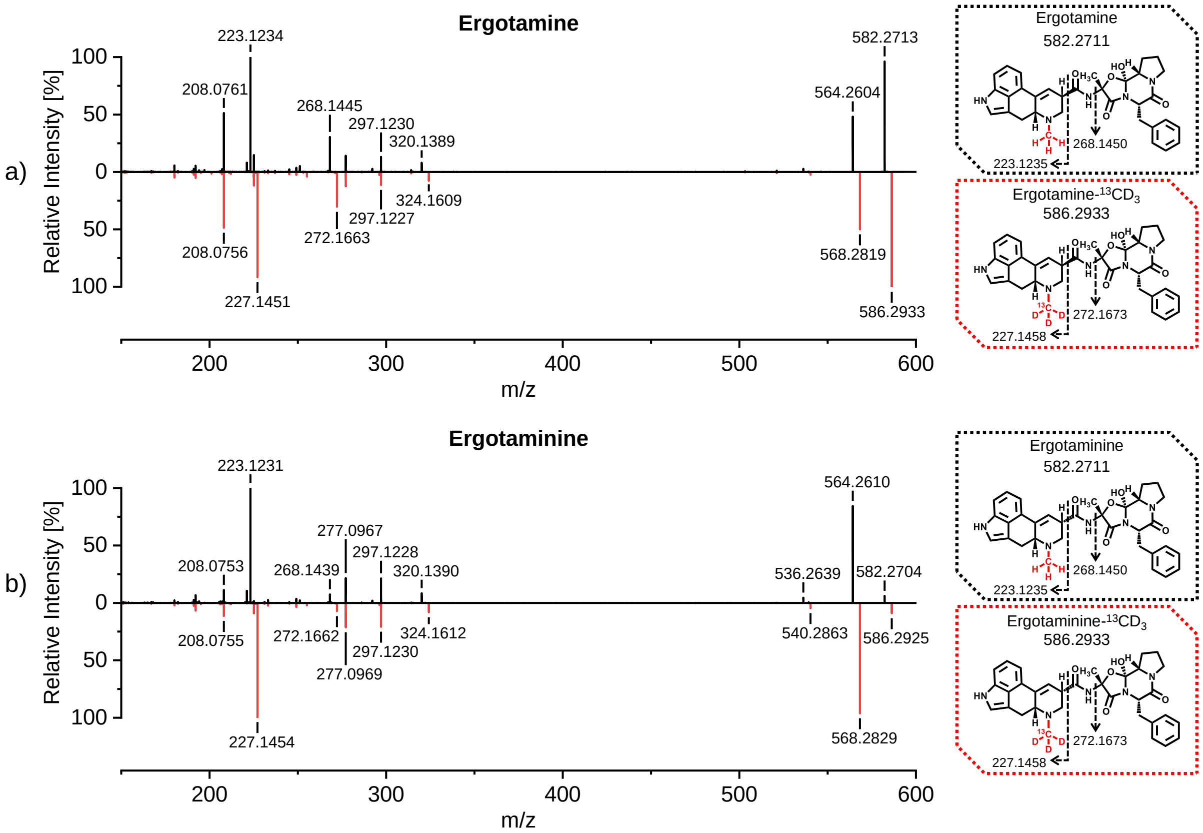

Figure S16. High resolution tandem mass spectra [M+H]$^+$ of a) native ergotamine (black) and isotopically labelled ergotamine-$^{13}CD_3$ (red) and b) native ergotaminine (black) and isotopically labeled ergotaminine-$^{13}CD_3$.

Table S14. Formula, exact *m/z* ratio, measured accurate *m/z* ratio and deviation Δm/m [ppm] for the main product ions [M+H]$^+$ of ergotamine-$^{13}CD_3$.

Formula	Theoretical *m/z* [M+H]$^+$	Measured *m/z* [M+H]$^+$	Δm/m [ppm]
$^{13}CC_{32}H_{32}D_3N_5O_5^+$	586.2933	586.2933	0.0
$^{13}CC_{32}H_{30}D_3N_5O_4^+$	568.2827	568.2819	-1.4
$^{13}CC_{18}H_{14}D_3N_3O_2^+$	324.1615	324.1609	-1.9
$C_{17}H_{16}N_2O_3^+$	297.1234	297.1227	-2.4
$^{13}CC_{15}H_{15}D_3N_3O^+$	272.1666	272.1663	-1.1
$^{13}CC_{14}H_{12}D_3N_2^+$	227.1452	227.1451	-0.4
$C_{14}H_{10}NO^+$	208.0757	208.0756	-0.5
